# Thermoelectric Characteristics of Bismuth Selenide Thin Films Prepared by Vacuum Thermal Evaporation of Bismuth Selenide Nanoparticles Synthesized by Hot Injection

**DOI:** 10.1021/acsomega.6c01937

**Published:** 2026-06-26

**Authors:** Claudia Patricia Villamizar Caballero, Angélica Lizbeth Espinosa Santana, Santhamma Maileppallil Thankamma Nair, Padmanabhan Karunakaran Nair

**Affiliations:** Instituto de Energías Renovables, 119952 Universidad Nacional Autónoma de México, Temixco, Morelos 62580, México

## Abstract

Bismuth selenide (Bi_2_Se_3_) thin films were deposited by vacuum thermal evaporation (VTE) of Bi_2_Se_3_ nanosheets (200 nm across, 20 nm in thickness), synthesized by the reaction of bismuth nitrate with selenourea in triethanolamine (TEA) applied via the hot injection method at 210 °C. Using 300 mg of nanosheets as the source material, thin films (70 nm thick) were produced on glass substrates held at 200 °C on a rotating substrate holder, with a source-to-substrate distance of 40 cm. Postdeposition heating of the thin films at 300 °C under nitrogen at 10 Torr, without or with the presence of elemental selenium powder, resulted in changes in the texture coefficient of the (006) planes of Bi_2_Se_3_ in its trigonal crystal structure (mineral: paraguanajuatite) from 5.5 (as-prepared film) to 6.8 (heated in nitrogen) and 8.2 (heated in nitrogen + selenium). Correspondingly, the electrical conductivity (σ) of the films increased from 2.5 to 2.9 × 10^3^ and 1.5 × 10^4^ (Ω·m)^−1^; the Seebeck coefficient (*S*, *n*-type) increased from 32 to 98 and 117 μV·K^–1^; and the thermoelectric power factor (σ·*S* ^2^) increased from 2.6 to 28 and 205 μW·m^–1^·K^–2^, respectively. A proof-of-concept five-element series-connected thin-film thermoelectric generator using the 45 or 70 nm Bi_2_Se_3_ thin film produced a voltage of 0.7 mV·K^–1^ and showed a response of 0.07 mV·s^–1^ or 0.1 K·s^–1^ when exposed to solar radiation (1000 W·m^–2^).

## Graphical abstract

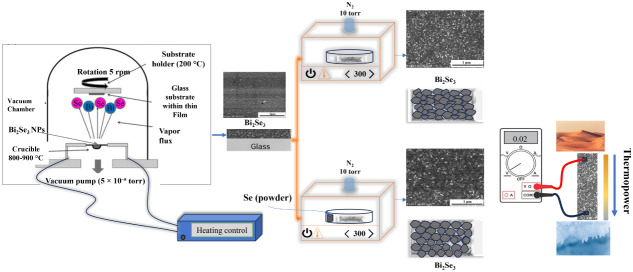

## 1 Introduction

Global energy and environmental challenges to reduce dependence on fossil fuels and lower environmental impacts require converting renewable heat sources into usable electrical energy.^1^ The direct conversion of thermal energy into electric energy in solid-state thermoelectric (TE) devices is recognized as a viable option for generating “green electricity” from industrial waste heat, solar thermal devices, geothermal energy, and body heat for wearable devices.^2^ In this context, nanostructured Bi_2_Se_3_ is among the promising thermoelectric materials due to its favorable electrical conductivity (σ) and Seebeck coefficient (*S*) at room temperature range (*T*, 280 to 320 K) that are comparable with those of established TE materials, Bi_2_Te_3_ and PbTe. An advantage of Bi_2_Se_3_ is that Se is relatively more abundant than tellurium in the Earth’s crust, which facilitates large-scale production of its TE devices. Other thermoelectric materials such as SnSe,^3^ Cu_2_Se,^4^ Sb_2_Te_3_,^5^ (Bi_*x*_Sb_1–*x*_)_2_Te_3_,^6^ and (Bi_1–*x*_Ni_*x*_)_2_Te_3_,^7^ have also been investigated due to their high performance near room temperature and their tunable transport properties, which are critical for enhancing the thermoelectric figure of merit.

In many cases, TE materials in thin film form show an enhanced thermoelectric figure of merit (*ZT* = *S* ^2^ σ *T*/κ), where κ is the thermal conductivity. The nanostructuring within the thin-film material induces quantum confinement effects that modify the electronic density of states near the Fermi level, thereby enhancing phonon scattering at grain boundaries and, in turn, reducing the lattice thermal conductivity.^8–10^ The power factor (*PF* = σ *S* ^2^) in the thin films may also be higher than that of their bulk materials. This occurs because grain-boundary-mediated scattering of minority charge carriers can suppress bipolar conduction, thereby yielding higher Seebeck coefficients in nanostructured systems than in their bulk counterparts.^11,12^ These quantum confinement effects are notable in one-dimensional (1D) structures such as nanowires and in two-dimensional (2D) materials.^13^ For this reason, there is growing interest in using deposition methods such as molecular beam epitaxy,^14,15^ chemical vapor deposition (CVD),^16^ pulsed laser deposition (PLD),^12^ vacuum thermal evaporation (VTE), and magnetron sputtering in order to get thin films of high crystallinity and oriented structures.^9,17,18^

Recent advances in defect engineering have highlighted the critical role of selenium vacancy compensation in improving the thermoelectric performance of Bi_2_Se_3_ films. In postdeposition thermal treatment of thin films, the ambient conditions during such treatment can significantly compensate for Se vacancies, reduce undesired bulk carrier density, and thereby enhance electronic transport.^19^ In such thermal processing, an ambient of Se helped modify the crystallographic orientation and carrier concentration in Bi–Se thin films, thereby enhancing their thermoelectric behavior.^19,20^ Optimization of the texture coefficient in the films, together with control of Se vacancies, has yielded marked improvements in the Seebeck coefficient and power factor for TE applications of these films.^11,20,21^ Specifically, the work on [00*l*]-oriented Bi_2_(Te, Se)_3_ thin films has shown that oriented growth combined with selenization improved their in-plane electrical conductivity and thermoelectric output, and provided a pathway for designing nanoscale TE devices.^13^ Studies on the thickness-dependence of charge carrier transport in Bi_2_Se_3_ thin films prepared by sputtering showed how quantum coherence and coupling between surface and bulk channels evolve with film thickness. These studies emphasized the importance of minimizing bulk conduction to maximize surface contribution.^11^

These findings collectively motivated the present methodology for growing nanostructured Bi_2_Se_3_ thin films by VTE using nanoparticle precursors, followed by annealing in a selenium-rich ambient to reconcile structural orientation and to compensate for vacancies. The synthesis and postdeposition thermal treatment of the materials played a crucial role in the development of the microstructure. Highly controlled growth of bismuth selenide VTE thin films, achieved through annealing these in a Se-ambient, improved their thermoelectric parameters in thin films with thicknesses of 45–70 nm.

## 2 Materials and Methods

### 2.1 Materials

Bismuth selenide in the form of nanosheets free from oxide content, used as the evaporation source in this work, was prepared in our laboratory, following the method reported previously.^22^ The following reagents were used in its preparation: selenium powder (99%, 100 mesh), benzimidazole (99%), iodomethane (99%), potassium carbonate (99%), Bi­(NO_3_)_3_·5H_2_O (98%), acetonitrile, methanol (98%), triethanolamine (TEA, 99%), and acetone. These reagents were supplied by Sigma-Aldrich.

### 2.2 Synthesis of Bismuth Selenide Nanosheets

Bismuth selenide (Bi_2_Se_3_) nanostructures were prepared by the hot injection method using 1,3-dimethyl-1*H*-benzo­[d]­imidazole-2­(3*H*)-selenone as the selenium precursor.^22^ For this, 0.287 g (0.59 mmol) of Bi­(NO_3_)_3_·5H_2_O was dissolved in 10 mL of triethanolamine (TEA) in a three-neck round-bottom flask. The solution was heated at 120 °C under vacuum for 1 h to remove dissolved oxygen. Separately, 0.2 g (0.89 mmol) of the selenone was suspended in 10 mL of TEA in a Schlenk flask, and the mixture was purged several times with argon, followed by a pump-down to produce a vacuum. The selenone suspension was then injected into the Bi­(III) solution, and the mixture was heated to 210 °C for 1 h. The solid form produced through the reaction was isolated from the reaction mixture via centrifugation. The product was washed several times with acetone. The reaction yield was 98%. Through various analyses, the product obtained by this methodology was determined to be Bi_2_Se_3_, with no oxide phase present. Hence, this product has the merit of serving as a reliable evaporation source for producing Bi_2_Se_3_ thin films by VTE for TE applications, as is described in Figure 1.

**Figure 1:**
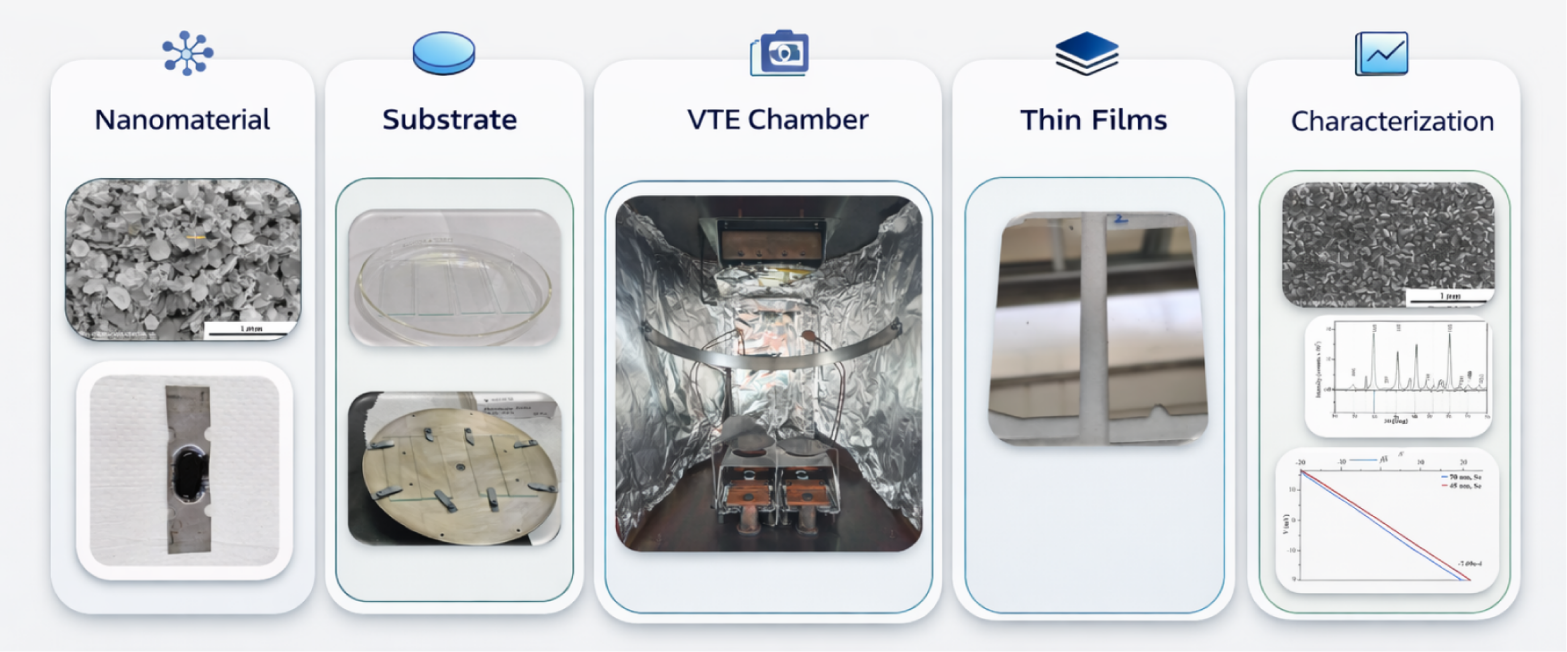
Schematic description for the preparation of bismuth selenide thin films by vacuum thermal evaporation using nanomaterials synthesized via the hot-injection method.

### 2.3 Deposition of Bi_2_Se_3_ Thin Film by Vacuum Thermal Evaporation (VTE)

A vacuum thermal evaporation unit (Torr International DP System), with substrate heating and a source-to-substrate distance of 40 cm, was used to deposit thin films of bismuth selenide. To begin with, six previously cleaned Corning microscope glass slides were supported on a stainless-steel disc substrate holder and clamped inside the VTE system. A motor was attached to the sample holder to rotate it during the deposition, ensuring uniform film thickness and composition. Tungsten-halogen lamps placed behind the rotating substrate holder maintained the substrate temperature by controlling the on–off duration of the lamps. A weighed quantity (100 mg, 200 mg, or 300 mg) of the Bi_2_Se_3_ nanosheets prepared as above was placed in a molybdenum crucible. A few drops of propylene glycol were added to it to facilitate the formation of a homogeneous paste. This paste was uniformly spread along the inner surface of the crucible and subsequently dried in an oven at 80 °C for 30 min to remove residual solvent. The crucible containing the material was clamped between two copper electrodes in the deposition chamber of the thermal evaporation system (Figure 1). The chamber pressure was reduced to 5 × 10^–5^ Torr using a pumping system comprising a mechanical pump and a diffusion pump. The substrate temperature was raised to and maintained at 200 °C. The sample holder rotated at 5 rpm throughout deposition. A direct current was applied across the crucible to increase the source temperature to approximately 800 °C–900 °C over a period of 5–10 min. The deposition procedure lasted for 30 min. This methodology was designed to improve the crystallinity and stoichiometry of the resulting thin films, 45 to 70 nm in thickness.

### 2.4 Heating of Thin Film Bi_2_Se_3_

The thin films were subjected to heat treatment at 300 °C for 30 min under a nitrogen atmosphere of 10 Torr, with and without the presence of 5 mg of elemental Se powder. Preliminary experiments have shown that a temperature of 300 °C and a duration of 30 min are optimal for thermal processing to improve the thermoelectric properties of the films.^20,23^ For the thermal treatment, with or without Se, the substrates coated with the films were placed in a Petri dish, which was then tightly wrapped with aluminum foil and placed in a vacuum furnace. For the heating in the presence of Se, 5 mg of Se powder (Sigma-Aldrich, 100 mesh) was taken in an aluminum foil basket and placed near the substrates in the Petri-dish. The heating was done at 300 °C for 30 min: (i) under a nitrogen pressure of 10 Torr, and (ii) at 300 °C for 30 min at a nitrogen pressure of 10 Torr in the presence of 5 mg of Se powder. The samples were heated to improve adherence and crystallinity, which could increase charge-carrier mobility and thus lead to higher electrical conductivity of the films. The addition of Se to the Petri dish was intended to convert any oxide present in the VTE thin film into bismuth selenide by providing a Se vapor ambient. The as-prepared and annealed films were characterized to determine their structural, optical, surface morphological, and electrical properties.

### 2.5 Characterization of the Films

The thickness of the films was measured on an Ambios Technology XP-200 unit. A Hitachi SU1510 Scanning electron microscope (SEM) was used to record the surface morphology of thin films. An energy-dispersive X-ray spectrometer (EDS) attached to the Hitachi SU1510 was used to determine the chemical composition of the films through the emission from the sample excited by a 15 keV electron beam incident perpendicular to the sample plane. In addition, a quantitative estimation of Bi and Se was also done using Inductively Coupled Plasma–Optical Emission Spectroscopy (ICP-OES) measurements in a HORIBA Scientific Ultima 2 ICP optical spectrometer. For this, the equipment was calibrated using an aqueous analytical solution prepared by diluting appropriate ratios of Bi and Se commercially available (Crescent Chemical Company) standard solution for element calibration that contains 1000 mg per liter of each element in 4% HNO_3_. The emission lines of Bi at 223.06 nm and of Se at 196.026 nm were used in the quantitative analyses under axial plasma observation. The ICP-OES spectrometer conditions are given in the Supporting Information file (Table S1). The optical transmittance (*T*) and specular reflectance (*R*) spectra of the films were recorded using air and a front aluminized mirror, respectively, as references in the 250–2500 nm spectral range on a Shimadzu 3600 UV–VIS-NIR spectrophotometer. A Thermo Scientific X-ray photoelectron spectroscopy (XPS) unit was used to record elemental surveys and binding energies of different atomic/ionic species in the films. The X-ray source was Al–Kα (Energy: 1486.68 eV, Voltage: 12 kV, Current: 3 × 10^–3^ A) with a microfocused X-ray monochromator. Detailed high-resolution spectra were acquired for the Bi-4*f*, Se-3*d,* and O-1*s* core-level regions. The films were also analyzed using depth profiling to investigate the distribution of different atomic species from the surface toward the depth of the film. For this, low-energy Ar-ion etching was performed in multiple cycles, each lasting 10–20 s, until the film–substrate interface was reached. After each etching step, high-resolution XPS spectra of the Bi-4*f* and Se-3*d* core-level binding energy regions were recorded. X-ray Diffraction (XRD) patterns for the structural characterization of Bi_2_Se_3_ nanomaterial and of the thin films were recorded on a Rigaku Ultima IV diffractometer with Cu–Kα radiation (λ = 1.5406 Å). A Jeol JEM-ARM200F scanning transmission electron microscope TEM/STEM was used to analyze the detailed structural and morphological properties of the materials. The Seebeck coefficient of the thin film was obtained from the voltage (V) measured across it as a function of the temperature difference ΔT, in the temperature interval 298 K–323 K. The differential temperature was provided by the heating/cooling produced across the thin film samples. The electrical properties, such as carrier concentration, mobility, resistivity, and conductivity type, were measured at room temperature using an HS-3000 Hall system in the Van der Pauw (VDP) geometry. The four-probe contact was made on silver paint placed at the corners of a square sample (1 cm × 1 cm) of the film deposited on glass substrates. For the Hall effect measurement, a magnetic induction of 0.5 T was provided by an electromagnet.

## 3 Results and Discussion

### 3.1 Thin Film Deposition by Vacuum Thermal Evaporation (VTE) of Bi_2_Se_3_ Nanosheets

The Bi_2_Se_3_ powder, in the form of nanosheets, used as the evaporation source, was free of oxygen with a Bi: Se ratio of 1:1.53 and a trigonal (rhombohedral) crystal structure, as indicated by the FESEM image, EDS, and XRD data (Figure S1a–c).^22^ The XRD pattern matches well the standard peaks reported in JCPDS No. 33-0214 for the bismuth selenide mineral, paraguanajuatite, with a trigonal (rhombohedral) crystal structure (space group R–3m), with *a* = 4.133 Å and *c* = 28.62 Å, consisting of three Bi_2_Se_3_ units per cell.^24^ The preparation of the evaporation source, as described in Section 2.3, might introduce some oxygen into the powder.

In VTE, material from a solid source is vaporized either as individual atoms (Bi, Se) or as molecular species (Bi_2_Se_3_) and transported through a vacuum or low-pressure gaseous environment (10^–6^–10^–5^ Torr) to a substrate maintained at a chosen temperature (200 °C in this case), usually below the melting point of the material deposited as thin film (706 °C for Bi_2_Se_3_). Upon reaching the cooler substrate surface, the vapor condenses, initiating nucleation and subsequent film growth. However, the deposited semiconductor thin film may differ from that of the source due to the prevailing conditions. Thus, postdeposition thermal treatment in a controlled ambient is a routine strategy to secure the desired properties for specific applications of the VTE thin films.

In this work, Bi_2_Se_3_ thin films of 45, 57, or 70 nm in thickness were obtained by varying the quantity as 100, 200, or 300 mg, respectively, of bismuth selenide powder placed in the Mo crucible as the source of evaporation. A variation in thickness proportional to the mass of the source material was not observed in these experiments. This would occur due to the solid–vapor phase equilibrium of Bi_2_Se_3_ during vacuum thermal evaporation, where the decomposition of the source into volatile Bi–Se and Se_2_ species governs the vapor flux.^25^ Under high vacuum and a substrate temperature of 200 °C, partial re-evaporation of volatile species further reduces the effective growth rate, leading to a nonlinear dependence of the film thickness on the source mass.^26^ Consistent with this, Bi_2_Se_3_ exhibits significant sublimation under vacuum below 280 °C, indicating enhanced volatile losses during film growth and thermal processing.^9,27^

### 3.2 Chemical Composition of Thin Films

Figure 2 shows the energy-dispersive X-ray spectroscopy (EDS) data for Bi and Se components in films with a thickness of 70 nm: as-deposited (top), and after heating at 300 °C for 30 min under 10 Torr nitrogen (middle), and 300 °C for 30 min under 10 Torr nitrogen with Se (bottom). The use of a 15 keV electron beam for this analysis causes emission from the constituent elements of the glass substrate, which contains about 71–75% SiO_2_, 12–16% Na_2_O, 8–16% CaO + MgO, 0–2% Al_2_O_3_ with K_α1_ emissions from O, Na, Mg, Si and Ca at 0.525, 1.041, 1.254, 1.740, and 3.692 keV, respectively, and Ca–K_β1_ emission at 4.013 keV. Handling of the sample involves carbon species with associated C–K_α1_ emission at 0.277 keV. The Bi-L_α1_ emission occurs at 10.839 keV, and the Se–K_α1_ emission at 11.222 keV, which are detected from the thin film. However, for the estimation of the Se/Bi atomic ratio in the thin film, the intensities of the dominant Se-L_α1,2_ emission at 1.379 keV and of the Bi-M_α1_ emission at 2.423 keV were chosen for the EDS analysis. This analysis shows a tendency of variation in the Se/Bi ratio as follows: 1.47, a value near the stoichiometric ratio 1.5 of Bi_2_Se_3_ for the as-deposited thin film; 1.40, which is a slight decrease in selenium content, likely due to selenium volatilization at elevated temperatures^23^ for the films annealed under nitrogen; and 1.57, which suggests incorporation of Se from the ambient for the films annealed under nitrogen with Se. According to some references, the improved Se/Bi ratio in the films influences their crystalline phase, which could modify their thermoelectric properties.^12,20^ Elemental mapping images (Figure S2a–d) confirm a homogeneous spatial distribution of Bi and Se and traces of O (Se–K_α1_, Bi-L_α1_, and O–K_α1_ signals are observed in the EDS, Figure S2e) on the surface of the films.

**Figure 2:**
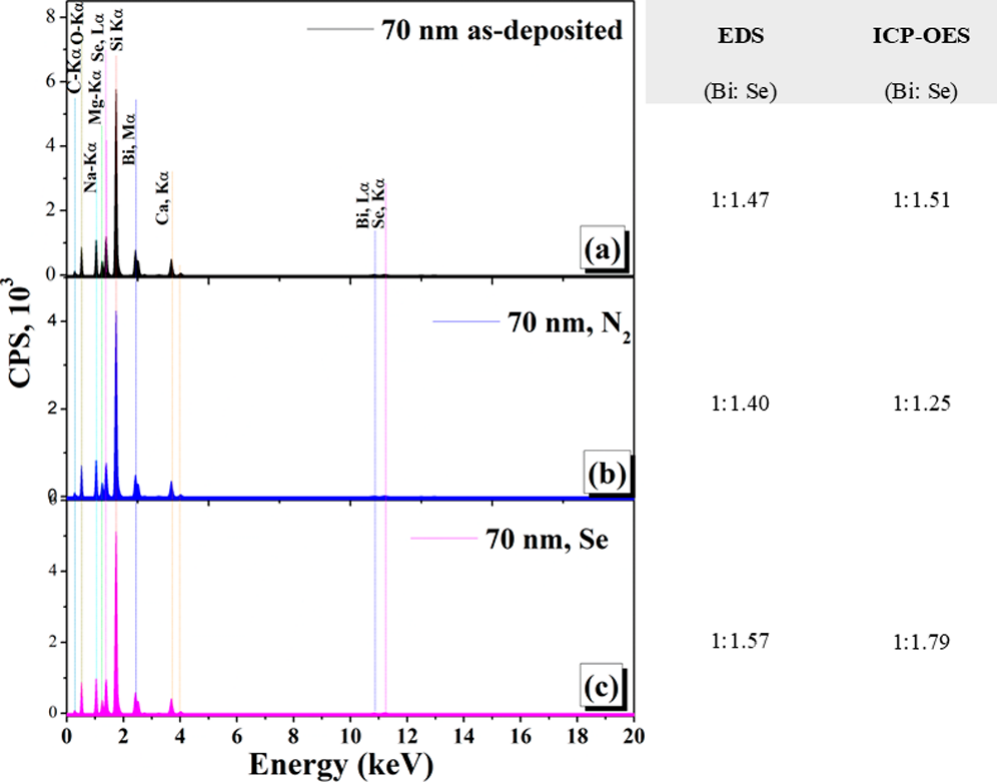
Intensity of X-ray emission peaks as a function of X-ray energy in the energy-dispersive X-ray spectra (EDS) of Bi_2_Se_3_ thin films (VTE, 70 nm thickness), as-deposited and after heating. The elemental composition and Bi:Se atomic ratios of the samples from EDS and ICP-OES analyses are given on the rightside panel.

The quantitative ICP-OES measurements also showed a similar trend in the variation of the Se/Bi ratio in the films. The analytical results for the samples (Table S2) and the calibration data (Figure S3) are presented in the Supporting Information file. Figure 2 gives a comparison of the results of analyses by the two techniques. Both techniques reveal the same overall compositional tendency associated with the thermal treatments, as described earlier.

Although the Se/Bi ratios differ between EDS and ICP-OES, the compositional trends are consistent. The differences between the two techniques can be attributed to their distinct analytical principles and sampling depths. EDS measurements in ultrathin (∼70 nm) films are affected by the relatively large electron-interaction volume generated by the 15 keV electron beam, which also excites emission from the glass substrate. Consequently, EDS should be considered semiquantitative under these conditions. In contrast, ICP-OES provides bulk quantitative elemental analysis of the completely dissolved thin film and therefore offers improved sensitivity and compositional accuracy. However, ICP-OES measurements represent an average composition across the entire dissolved film and do not provide information on possible compositional gradients through the film thickness.

The XPS survey spectra of the as-prepared and heated films (Figures S4–S6a) showed photoelectron peaks corresponding to Bi (4*p*, 4*d*, 4*f*, 5*d*), Se (3*p*, 3*d*), C (1*s*), and O (1*s*). The carbon 1*s* peak observed at the binding energy 284.8 eV arises from hydrocarbon contaminants on the surface.^28^ The appearance of the Si 2*p* peak at 103.3 eV after 20 s of Ar-ion etching of the film confirms the proximity of the film-glass substrate (SiO_2_) interface at an etch rate of 2 nm s^–1^.

The XPS depth profile analyses of the as-prepared films and those annealed under 10 Torr nitrogen with or without the presence of Se (Figures S4–S6b,c) show that the intensities of both Bi-4*f* and Se-3*d* peaks remain without variation in the spectra recorded after 20 s of etching of the films. This indicates that the thin films exhibit a homogeneous chemical composition (Bi: Se) profile throughout their thickness. The high-resolution XPS spectra presented in Figure 3a, b show Bi-4*f* and Se-3*d* peaks deconvoluted. The peaks at binding energies of 157.38 and 162.68 eV in the spectra of the as-deposited films correspond to Bi 4f_7/2_ and Bi 4*f*_5/2_, respectively, with a spin–orbit splitting of 5.3 eV. These values are consistent with literature-reported binding energies for Bi^3+^ species.^28^

**Figure 3:**
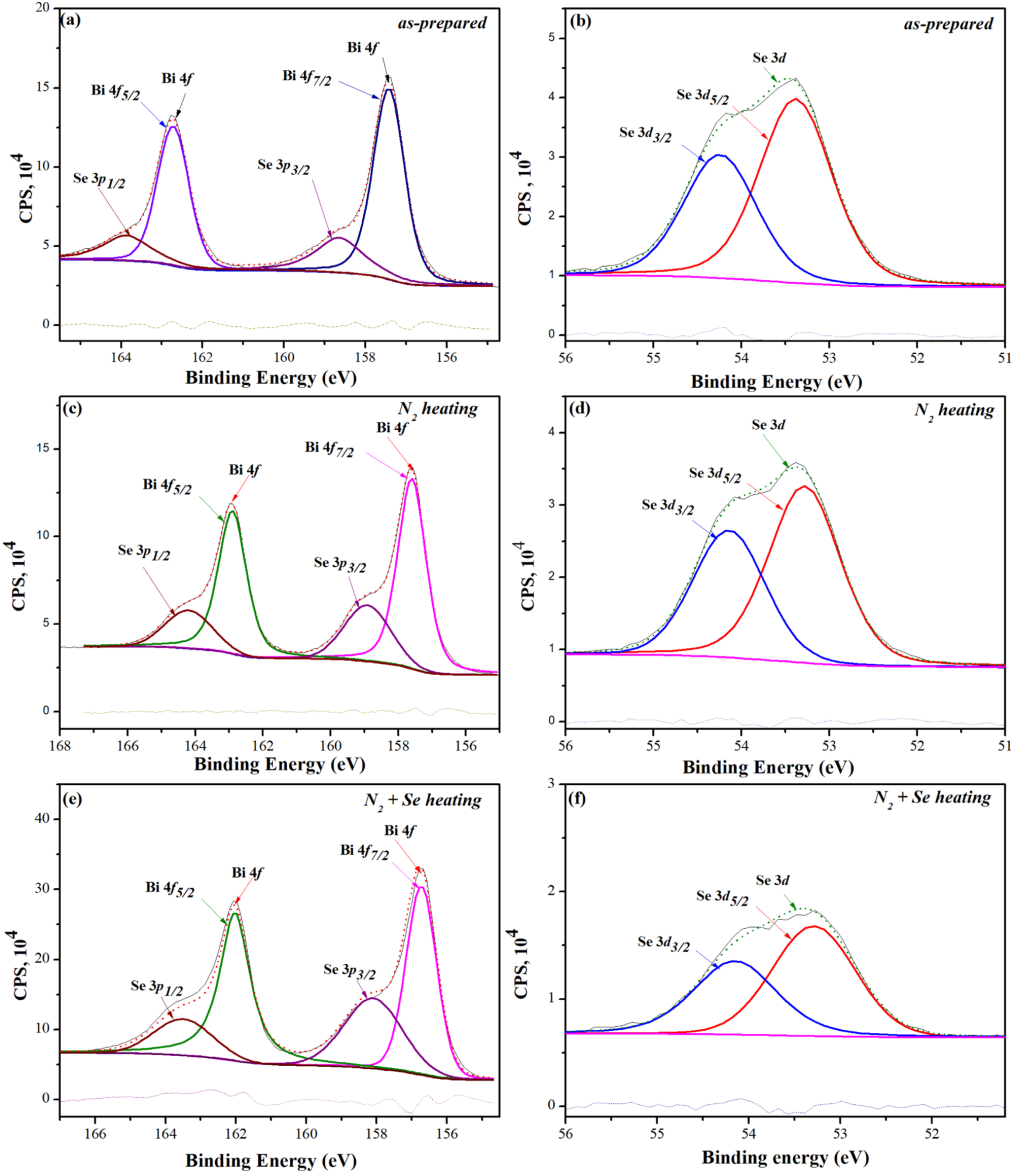
XPS high-resolution spectra of bismuth selenide VTE thin film showing the binding energies corresponding to the Bi 4*f* and Se 3*d* states, respectively: (a, b) as deposited; (c, d) for the film annealed in nitrogen atmosphere; and (e, f) for that annealed in nitrogen with Se vapor.

In addition, two less intense peaks were observed at 158.68 and 163.88 eV, assigned to the Se 3*p*_3/2_ and Se 3*p*_1/2_, respectively.^29^ The Se-3*d* spectrum is resolved into two peaks at 53.28 eV (Se-3*d*_5/2_) and 54.28 eV (Se-3*d*_3/2_), with a spin–orbit splitting of 0.9 eV. According to literature values, elemental selenium (Se^0^) shows binding energies of 54.7 eV (3*d*_5/2_) and 55.1 eV (3*d*_3/2_), while selenide species (Se^2–^) exhibit binding energies below 55 eV.^30^

In the case of the thin film annealed in a nitrogen ambient (Figure 3c,d), the Bi-4*f* region exhibited two deconvoluted peaks at binding energies 162.08 eV (Bi^0^ 4*f*_5/2_) and 156.78 eV (Bi^0^ 4*f*_7/2_), as well as 163.58 eV (Bi^3+^ 4*f*_5/2_) and 158.08 eV (Bi^3+^ 4*f*_7/2_) with a spin–orbit splitting of 5.2 eV. The Se-3*d* region was deconvoluted into two peaks at 53.28 eV (Se^2–^ 3*d*_5/2_) and 54.18 eV (Se^2–^ 3*d*_3/2_) corresponding to a spin–orbit splitting of 0.9 eV. The presence of Bi^0^ species is attributed to the formation of Bi–Bi bonds resulting from selenium volatilization at elevated temperatures.^20,23^

In the high-resolution Se-3*d* and Bi-4*f* spectra for the thin films annealed in a selenium-rich ambient (Figure 3d, e), photoelectron peaks were observed at binding energies 162.88 eV (Bi^3+^ 4*f*_5/2_), 157.58 eV (Bi^3+^ 4*f*_7/2_), 164.18 eV (Se^2–^ 3*p*_1/2_), and 158.98 eV (Se^2–^ 3*p*_3/2_). The peaks located at binding energies of 53.28 and 54.18 eV, respectively, correspond to the Se 3*d*_5/2_ and Se 3*d*_3/2_. It is also notable that no characteristic signal corresponding to elemental selenium (Se with binding energy 55.1 eV) was observed in the spectra of any of the films.

### 3.3 Morphology and Crystal Structure of the Bi_2_Se_3_ Thin Films

Low-magnification scanning electron microscopy (SEM) was used to investigate the microstructure of the Bi_2_Se_3_ thin films. The as-deposited films had a smooth, mirror-like surface with a metallic shine. The micrographs in Figure 4a–c show that the bismuth selenide films with 70 nm in thickness are composed of regular, smooth, and uniformly sized particles. Thin films annealed in a selenium-rich atmosphere displayed a nanoflake morphology on the surface, with an average size of approximately 10.3 nm. In the cross-sectional SEM images given in Figure 4d–f, the micrograph (f) shows a preferential perpendicular growth of these nanoflakes relative to the substrate. The micrographs of both as-deposited and annealed thin films, with thicknesses of 45 and 57 nm (Figures S7a–f), showed that morphology depends on both the thickness and the annealing conditions.^11,31^

**Figure 4:**
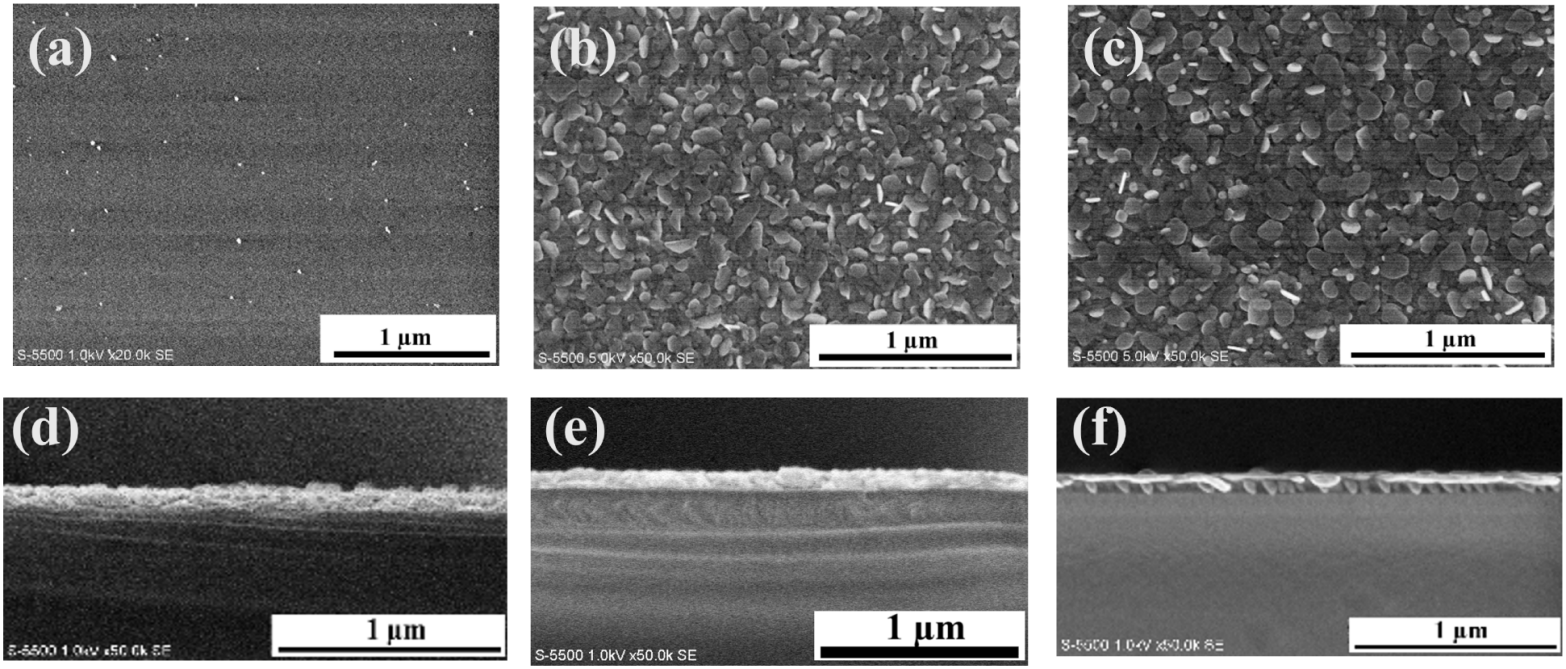
**SEM** images of the Bi_2_Se_3_ films: (a) as-deposited, (b) annealed in N_2_, and (c) Se + N_2_ atmosphere, (d–f) cross-sectional images of thin films of 70 nm in thickness.

This morphology was further confirmed by TEM images (Figure S8a–c), which showed that the films predominantly grow along the *c*-axis, forming a layered structure composed of oriented grains within each layer. In this layered morphology, five atomic layers arranged in the sequence – Se–Bi–Se–Bi–Se– form the basic unit, known as a quintuple layer (QL), which interacts with adjacent layers through van der Waals forces.^15,16^

The grazing incidence X-ray diffraction (GIXRD) patterns of the thin films (70 nm), as-deposited and after the heating under nitrogen without and with selenium rich atmosphere, presented in Figure 5 show peaks that match the standard peaks reported in the JCPDS file No. 33-0214 for the mineral paraguanajuatite consisting of Bi_2_Se_3_ with a trigonal (rhombohedral) crystal structure (space group *R*3̅*m*; *a* = *b* = 4.1396 Å and *c* = 28.6360 Å). No peaks attributable to impurities were detected, indicating that the polycrystalline thin film is of Bi_2_Se_3_.^9^ A particularly strong diffraction peak is seen at 2θ = 18.4°, corresponding to the diffraction from (006) planes, suggesting that crystal growth is predominantly oriented along the *c*-axis [*00l*]. The texture coefficient (*TC*_(*hkl*)_) of the Bi_2_Se_3_ film was calculated from the XRD peaks to determine the preferential crystallographic orientation using eq 1.^32^

**Figure 5:**
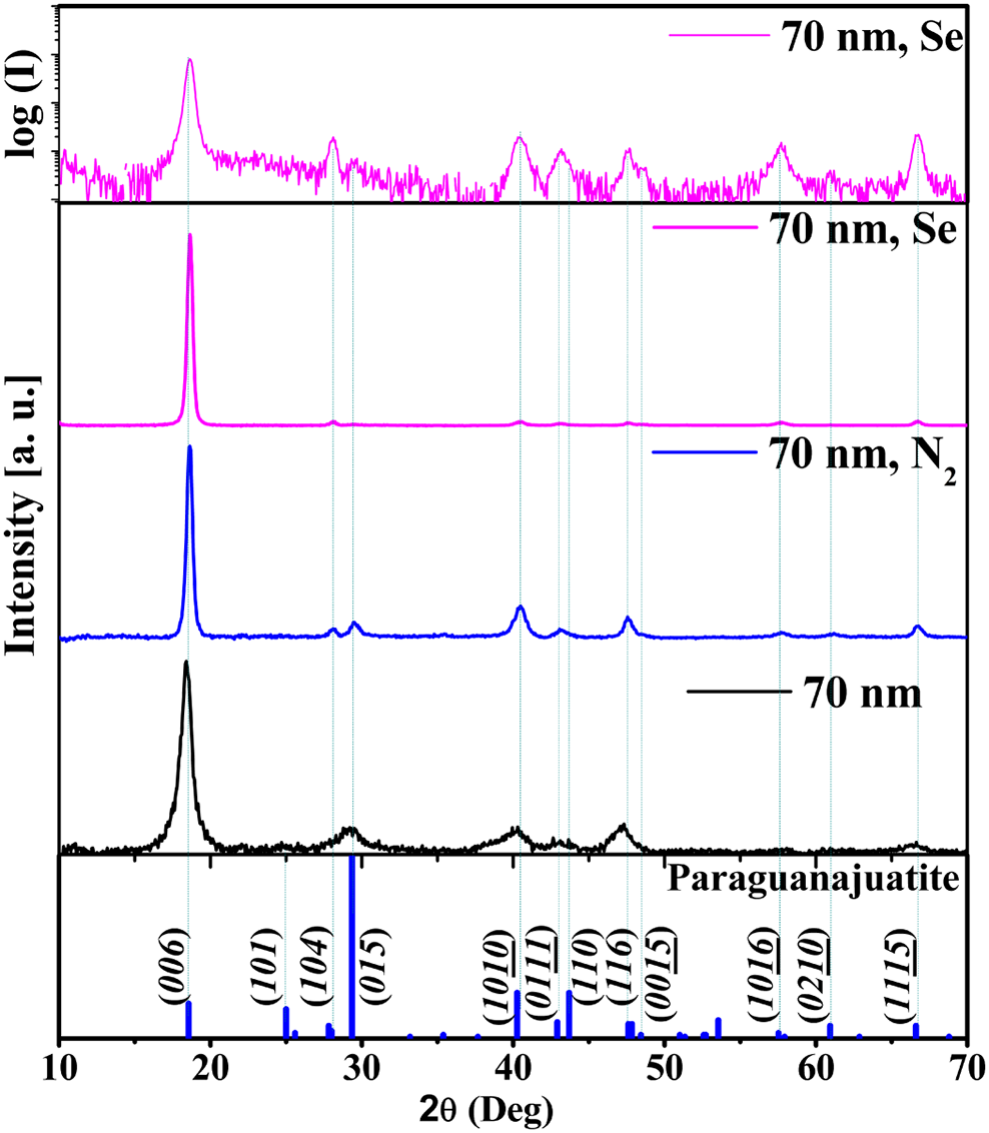
GIXRD patterns, recorded using a grazing angle of 0.5°, of the as-deposited and heated (under 10 Torr nitrogen without and with 5 mg Se) thin films of Bi_2_Se_3_ (70 nm) deposited by VTE on glass substrates. Top panel, log-intensity plot of the pattern labeled 70 nm, Se; Bottom panel, the standard pattern (PDF#00-033-0214) of paraguanajuatite mineral.


1 $$TC_{\left(hkl\right)}=\frac{I_{\left(hkl\right)}}{I_{0\left(hkl\right)}}/\frac{1}{N}\left[\overset{N}{\underset{i=1}{\sum }}\frac{I_{\left(hkl\right)}}{I_{0\left(hkl\right)}}\right]$$

Here, *I*_(*hkl*)_ is the measured intensity of the peak corresponding to a given plane, *I*_0(*hkl*)_ corresponds to the intensity of the diffraction peak from that plane in the powder diffraction file, and *N* is the number of XRD peaks considered in the calculation. The *TC* values for the (006) planes are greater than 5, confirming a pronounced texture for the material growing with these planes parallel to the substrate surface and perpendicular to the *c*-axis (Figure S9a).^33^ Thus, the films exhibit a strongly preferred orientation perpendicular to the *c*-axis [006]. Films of 45 and 57 nm in thickness also show similar XRD patterns (Figure S9b–c), all with a texture coefficient (*TC*) above 5 for the (006) plane.

The average crystallite size (*D*) of the Bi_2_Se_3_ thin films was estimated using the Scherrer formula, eq 2,^34^ for spherical particles:

2 D=0.94λ/[βcos(θ)]

Here, *D* is the average crystallite size, β is the full width at half-maximum (FWHM) of the diffraction peak in radians, λ is the X-ray wavelength (1.5406 Å), and θ is the Bragg angle. This estimation does not account for contributions from lattice strain or structural defects, which may broaden the peaks. The crystallite sizes of Bi_2_Se_3_ thin films ranged from approximately 77 to 174 Å (7.7 to 17.4 nm). Upon annealing, the crystallite size increased, consistent with improved crystallinity. The films heated with Se exhibit a larger crystallite size. During annealing, selenium powder sublimes and diffuses throughout the film, reacting with the traces of bismuth to form Bi_2_Se_3_.^20^ The X-ray diffraction (XRD) patterns of all samples showed no peaks corresponding to bismuth oxide or other secondary phases such as elemental selenium.

The Williamson–Hall (W–H) method has also been applied to estimate crystallite size using eq 3,^35,36^ which is more accurate because it accounts for broadening caused by crystallite size and lattice strain. Furthermore, it provides information about the nature of the strain (tensile or compressive). In the present case, the W–H analysis shows good agreement with the Scherrer results, confirming the reliability of the observed trend as seen in Figure S10 and Table S3.

3 $$\beta \;\cos\left(\theta \right)=\frac{0.94\lambda }{D}+4\varepsilon \;\sin\left(\theta \right)$$

The microstrain (ε) was calculated from the W–H plots,^37^ and the data are summarized in Table S4. The as-deposited film shows a notable microstrain of (7.71 ± 2.3) × 10^–2^, indicating lattice distortion and internal stress. Following the heat treatment, the strain decreases considerably to (3.5 ± 1.21) × 10^–2^ for the N_2_-treated sample and to approximately (∼3.0 ± 0.95) × 10^–2^ for the Se-treated films. This reduction indicates effective relaxation of internal stresses and improved lattice uniformity. The reduced strain in Se-treated samples suggests that selenium incorporation during annealing with Se enhances structural ordering more effectively than annealing under N_2_ alone.

The dislocation density (δ) was calculated as (1/*D* ^2^),^38^ which represents the density of crystallographic defects and was found to decrease significantly after the heat treatments. The as-deposited film exhibits a relatively high dislocation density of (3.9 ± 1.5) × 10^– 4^ nm^– 2^. In contrast, the heat-treated films show values in the order of 10^–5^ nm^–2^, with the lowest value (3.4 ± 0.8 × 10^–5^ nm^–2^) observed for the Se-treated 70 nm film. This reduction is attributed to grain growth (from 7.7 to 17.4 nm, Table S3) and to defect reduction during postdeposition treatment.

The stacking fault (*SF*) can be determined using eq 4,^37,38^

4 $$SF=\left[\frac{2\pi^{2}}{45\sqrt{(3\tan\left(\theta \right)}}\right]\left(FWHM\right)$$

The stacking fault (*SF*) provides insight into planar defects within the crystal structure. The as-deposited film has an *SF* of (5.65 ± 0.87) × 10^–3^. The *SF* decreases to (3.1 ± 0.7) × 10^–3^ in the N_2_-treated sample and further to (2.3–2.6) × 10^–3^ in the Se-treated films. This reduction confirms improved structural ordering and reduced defect concentration.

The high-resolution transmission electron microscopy (HRTEM) image of a homogeneous thin region of a flake from the annealed film is shown in Figure 6a. The image shows lattice fringes corresponding to a polycrystalline thin film with interplanar spacings of 0.322, 0.274, and 0.305 nm. The selected-area electron diffraction (SAED) pattern, Figure 6b, confirms a polycrystalline structure, exhibiting diffraction spots indexed to the (101), (009), (0012), (0111), (110), (205), and (0210) crystalline planes. A summary of the data obtained from XRD, HRTEM, and SAED analyses is provided in Table S5.

**Figure 6:**
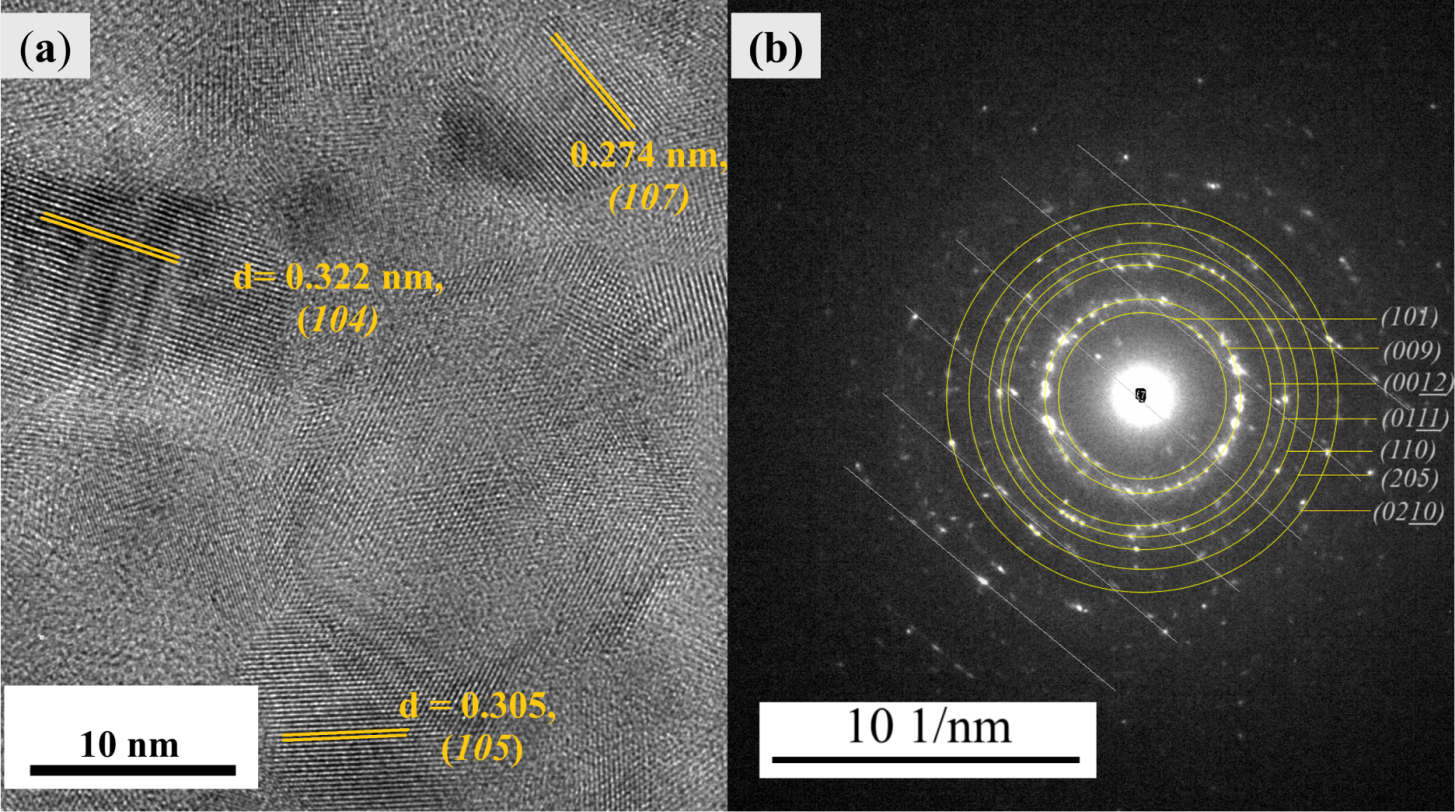
HRTEM images of (a) Bi_2_Se_3_ thin film annealed in a Se-rich atmosphere (70 nm), (b) SAED pattern.

Thus, the thin film of 70 nm in thickness deposited on a glass substrate by VTE of the nanoflake powder source synthesized in the laboratory by the hot injection method is Bi_2_Se_3_ with a trigonal (rhombohedral) crystal structure. Upon heating the films in the presence of Se, the crystallite diameter increases from 7.7 nm (as-deposited) to 17.4 nm, with the texture coefficient increasing from 5 to 8.2 for the *(006)* plane. This indicates that Bi_2_Se_3_ grows preferentially with the *(00l*) planes parallel to the glass substrate, with the *c*-axis of its crystallites oriented perpendicular to the substrate plane. This is accompanied by a notable improvement in its surface morphology (Figure 4).

### 3.4 Optical Characterization

Bismuth selenide is a known topological insulator.^10^ The optical behavior of its thin film is strongly influenced by the atomic structure, chemical composition, and bonding characteristics of the material. These factors define the optical properties and the band gap (*E*_*g*_).^39^ The optical transmittance (*T*) and reflectance (*R*) spectra measured in the wavelength range of 250–2500 nm of the bismuth selenide thin films of 70 nm thickness are given in Figure 7 along with the *T* + *R* plot.

**Figure 7:**
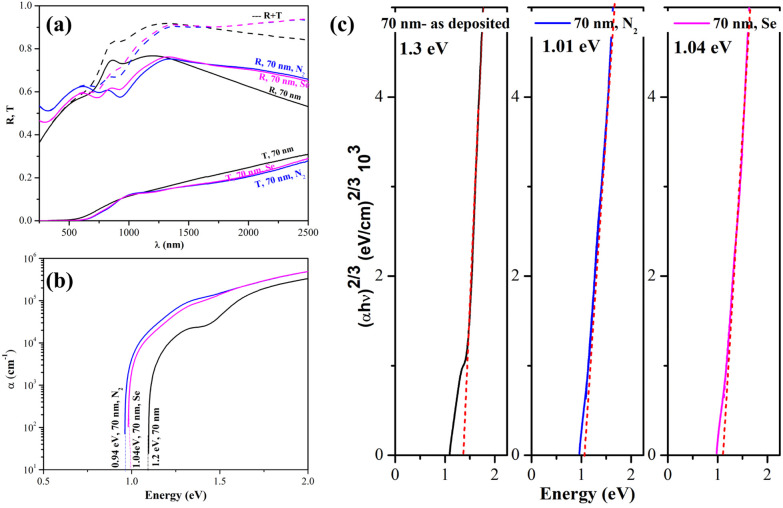
(a) Optical reflectance (*R*) and optical transmittance (*T*) of Bi_2_Se_3_ thin films of thickness 70 nm deposited by vacuum thermal evaporation; (b) optical absorption coefficient (α); and (c) plots of (αhν)^2/3^ against photon energy (hν).

The reflectance and transmittance of the films vary with film thickness and annealing conditions. The optical spectral data for films of different thicknesses are shown in Figure S11. For all these films, the sum of *T* + *R* remains close to 0.9–1 at wavelengths (λ) > 1000 nm, where optical absorption does not occur in the films. The average reflectance of the as-prepared thin film is 0.75, while that of the films obtained after heating is 0.65. The refractive index of the material of the film is defined by eq 5.^40–42^

5 $$n=\frac{\left(1+\sqrt{R}\right)}{\left(1-\sqrt{R}\right)}$$

Thus, its value is 13–13.9 for the as-deposited film and 9–9.32 for the heated films. The squares of these values, 169 and 81, respectively, give the high-frequency relative permittivity (ε_r∞_) of the materials. These are considerably larger than the cited values for these, 17.5–29,^43^ but are comparable with the cited static relative permittivity (ε_r∞_) of 113 for Bi_2_Se_3_ crystals. To explain the disparity, it is useful to consider that these thin films exhibit a high *TC* of 5.1–8.2 when propagating along the *c*-axis.

The optical absorption coefficient (α) was calculated from optical transmittance and reflectance data, accounting for multiple internal reflections, using eq 6.^39,40^

6 $$\alpha =-\frac{1}{d}\ln\left(\frac{\sqrt{{\left(1-R\right)}^{4}+{\left(2\cdot T\cdot R\right)}^{2}-{\left(1-R\right)}^{2}}}{2\cdot T\cdot R^{2}}\right)$$

These data are given in Figure 7b, showing the variation of α as a function of photon energy (hν). The film exhibits optical absorption coefficients of the order of 10^5^ cm^–1^, consistent with previously reported values for Bi_2_Se_3_.^44^ The optical bandgap energy (*E*_*g*_) values were determined using the Tauc relation, eq 7:^37^

7 $$(\alpha h\upsilon {)}^{n}=A\left(h\upsilon -E_{g}\right)$$

Figure 7c shows that a straight line with a correlation coefficient greater than 0.999 is obtained for n = 2/3. This suggests that the absorbed photons give rise to “forbidden transitions” across a direct bandgap of nearly 1 eV.^40^ This is considerably higher than the band gap *E*_*g*_ of 0.16 eV of the Bi_2_Se_3_ crystal.^43^ Thin films of Bi_2_Se_3_ are reported to show higher *E*_*g*_ values due to the quantum confinement effect of excitons with effective masses of electrons (*m*_*n*_) and holes (*m*_*p*_) averaged at 0.125 m_e_.^43^ The reduced mass of the exciton (*m*_*ex*_) in Bi_2_Se_3_ is 0.0625 m_e_, where m_e_ is the free-electron mass, 9.11 × 10^–31^ kg. From the Bohr model, the exciton radius is 0.053 nm × [113/(*m*_*ex*_/*m*_*e*_)], which is 96 nm.^40^ Thus, with a crystalline diameter (D) of 7.7–17.4 nm in the films, as evidenced in the XRD, strong quantum confinement of the exciton would occur. The increase in bandgap (eV) is 3/[(*D* nm)^2^ (*m*_*ex*_ */m*_*e*_)]. For the as-deposited VTE film of 70 nm in thickness with *D* = 7.7 nm, the increase in the bandgap is by 0.81 eV, which, when added to the crystal *E*_*g*_ of 0.16 eV, gives a value of 0.97 eV for the polycrystalline VTE thin films of Bi_2_Se_3_. This is approximately the value observed for the present films, as shown in Figure 7c. Figure S11c and f illustrates the extracted bandgap values for the films of different thicknesses, both for the as-deposited by VTE as well as the films after the heating.

### 3.5 Electrical and Thermoelectric Characteristics

A negative Hall coefficient was obtained for all the Bi_2_Se_3_ thin films—the as-deposited VTE films as well as for the films heated in nitrogen and in a selenium-rich ambient. From the *n-*type conductive characteristics, the electron concentration and Hall mobility (μ_nH_, taken approximately as the drift mobility μ_ndr_) were obtained. The majority carriers in films or crystals are electrons originating from selenium vacancies (V_Se_). The results of Hall-effect measurements on the films are summarized in Table 1, along with parameters obtained from thermoelectric measurements shown in Figure 9. The values of carrier concentration (*n*_*n*_), mobility (μ_*nH*_), and electrical conductivity (σ_*n*_) refer to values obtained from measurements made at room temperature, taken as 300 K. The Seebeck coefficients (*S*) and thermoelectric Power Factor (*PF*) values also refer to the room temperature range, 300 ± 30 K, relevant for wearable TE devices.

**Table 1:** Carrier Concentration (*n_n_*), Hall Mobility (μ_nH_), and Electrical Conductivity (σ_n_) Values Obtained from Hall Measurements at Room Temperature^a^

Sample	*n*_*n*_ (cm^–3^)	μ_*nH*_ (cm^2^/V**·**s)	*σ*_*n*_ (Ω**·**m)^−1^	*S* **(μV·K** ^– 1^)	*PF* = *S* ^2^·σ(μW**·**m^–1^ **·**K^– 2^)
45 nm VTE	4.8 × 10^19^	4.8	1.9 × 10^3^	–36	2.4
57 nm VTE	6.4 × 10^19^	5.5	5.6 × 10^2^	–77	3.3
70 nm VTE	4.3 × 10^19^	3.7	2.5 × 10^3^	–32	2.6
Crystal^43^	1.8 × 10^19^	690	-	-	-
45 nm, N_2_	1.60 × 10^15^	288.8	7.41 × 10^2^	–131	1.2
57 nm, N_2_	4.78 × 10^15^	1040	6.14 × 10^3^	–92	51
70 nm, N_2_	1.67 × 10^18^	1107	2.96 × 10^3^	–98	28
45 nm, Se	4.5 × 10^19^	2.2	1.6 × 10^3^	–138	30
57 nm, Se	1.6 × 10^19^	16.9	3.4 × 10^3^	–156	83
70 nm, Se	**2.37 × 10** ^**20**^	**23.5**	**1.50 × 10** ^**4**^	**–117**	**206**

a Seebeck coefficient (S) was obtained separately, and the thermoelectric power factor (PF = S²·σ n) was determined.

The carrier concentration (*n*_*n*_) in thin Bi_2_Se_3_ films decreased notably after their annealing in a nitrogen atmosphere at 300 °C for 30 min, as indicated by a substantial reduction in n_n_. This decrease is attributed to the passivation or elimination of Se (Group VI)—vacancies, which otherwise act as donor defects in Bi_2_Se_3_. Annealing in an inert nitrogen (Group V) environment likely facilitates defect rearrangement and stoichiometric stabilization. Specifically, the substitution of a Group V element at the Group VI vacancy reduces the *n*-type character and can deplete the electron concentration. However, the mobility (μ_*nH*_) increases significantly, from 288.8 to 1107 cm^2^ /(V·s) in these defect-compensated Bi_2_Se_3_ films. The reduction in carrier concentration in the films upon heating will, in turn, result in higher Seebeck coefficients for the films than those for the VTE films before heating, according to the relation *S* = (k_B_/q) ln­(*N*_*c*_/n_n_) = 86 μV·K^– 1^ ln­(N_*c*_/ n_n_).^45^ Here, k_B_ is the Boltzmann constant (1.380649 × 10^–23^ J K^–1^), q is the electron charge (1.602 × 10^–19^ C), and N_*c*_ is the density of state factor for the conduction band (m^–3^ or cm^–3^ at 300 K) of the semiconductor. In contrast, samples annealed at 300 °C in a selenium-rich atmosphere in general exhibit an increased carrier concentration relative to the as-deposited films, consistent with previous reports. The magnitude of this increase depends on the film thickness. This behavior is primarily ascribed to the selenium-rich treatment, which enhances the *c*-axis orientation and the average grain size of the films, as evidenced in the XRD results. As the carrier concentration is directly proportional to the crystalline grain diameter and inversely proportional to the grain boundary barrier height, the films with a high degree of preferential orientation exhibit smaller grain boundary inclination angles, leading to reduced grain boundary barriers and enhanced in-plane carrier mobility.^11,20^ The heat treatment in a selenium-rich atmosphere induced grain growth, in which individual grains merge and enlarge, resulting in a larger grain size (from 7.7 to 17.4 nm) and a more uniform film surface.

The granular structure in the film improves its thermoelectric properties. When the grain size is small and well-dispersed, the scattering of phonons occurs at the grain boundaries. This results in a decrease in thermal conductivity that would increase the thermoelectric figure of merit, which is inversely proportional to the sum of the free-carrier (κ_*e*_) and lattice (κ_*l*_) contributions to the thermal conductivity. For Bi_2_Se_3_ crystals, κ_*e*_ of 2.5 W m^–1^ K^–1^ and κ_*l*_ of 1.34 W m^–1^ K^–1^ have been reported.^43^ Thus, a notable reduction in κ_*l*_ is beneficial. When this reduction coincides with an increase in *S*, the power factor (*PF*) improves.^46^ Figure 8 shows that, for all thicknesses of VTE Bi_2_Se_3_ thin films, heating in a Se ambient or in an N_2_ only ambient increases the value of *S* (Table 1).

**Figure 8:**
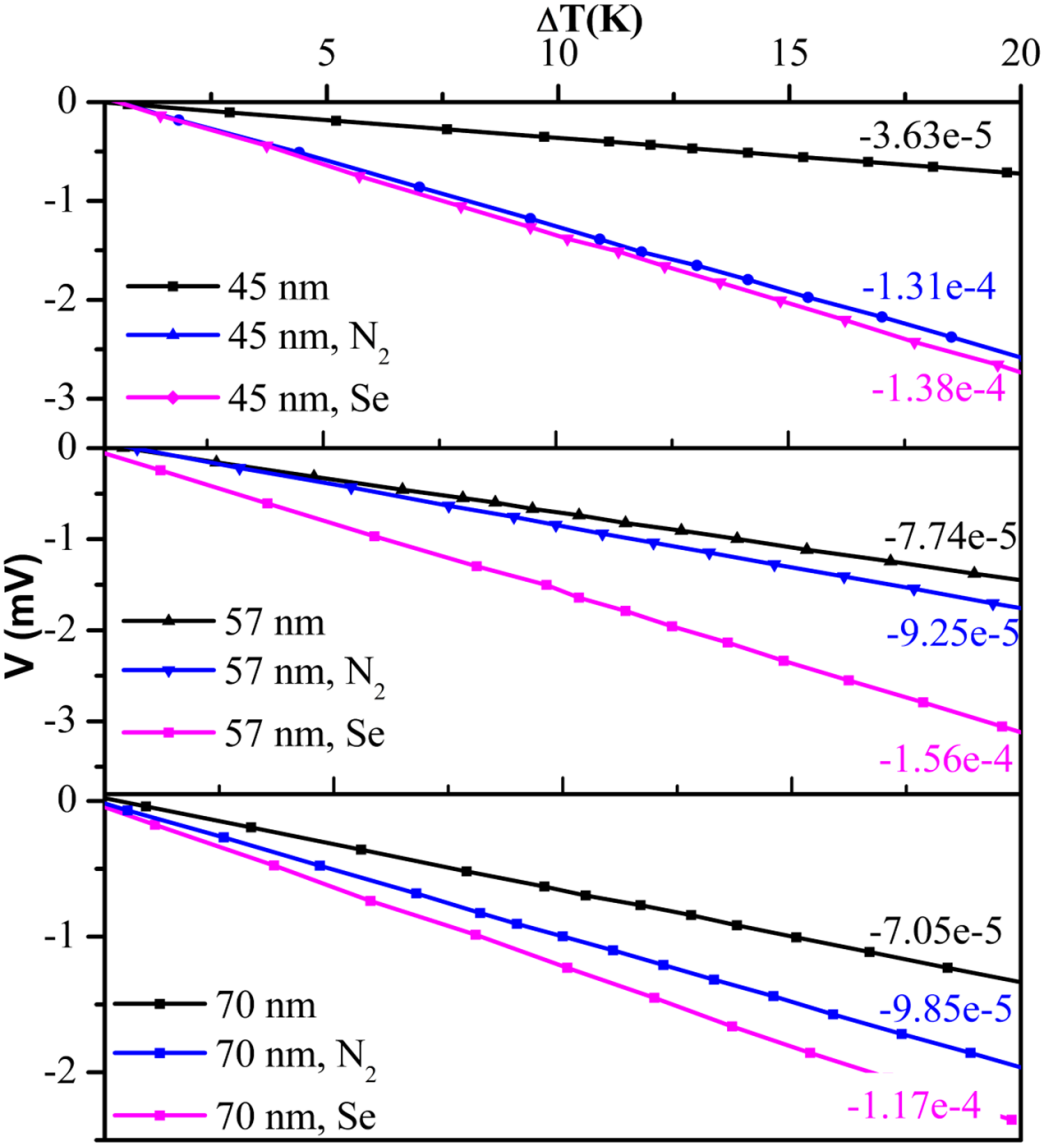
Seebeck coefficient (shown at right in V**·**K^– 1^) of bismuth selenide thin films of thicknesses 45, 57, and 70 nm produced by VTE of laboratory-synthesized nanoflake powder: as deposited, annealed in nitrogen atmosphere (N_2_), and in a selenium-enriched N_2_ atmosphere. ΔT is in the temperature interval of 298 K–323 K.

### 3.6 Improved Thermoelectric Characteristics of Annealed VTE Bismuth Selenide Thin Films

The enhanced thermoelectric performance of the annealed Bi_2_Se_3_ thin films prepared by VTE originates from the modification of the microstructure of the thin film: crystallographic texture, grain size, and the number of selenium vacancies. The use of phase-pure, hot-injection-derived Bi_2_Se_3_ nanoparticles as the evaporation source minimized oxide-related defects, enabling effective postdeposition structural tuning.^22^

The as-deposited films exhibit a strong (*00l*) preferential orientation with *TC*(006) ≈ 5.5. Annealing in N_2_ slightly increases the texture coefficient to 6.8, whereas annealing in N_2_ + Se vapor significantly enhances it to 8.2, indicating nearly complete alignment of quintuple layers parallel to the substrate. Because charge transport in Bi_2_Se_3_ is anisotropic and favored within the quintuple layers, this increased (*00l*) texture directly promotes in-plane carrier mobility. Simultaneously, annealing in a Se-rich atmosphere increases the crystallite size from 7.7 to 17.6 nm, reducing grain-boundary scattering. For the 70 nm films, Hall mobility increases from 3.7 to 23.5 cm^2^ V^–1^ s^–1^, and electrical conductivity increases from 2.5 × 10^3^ to 1.5 × 10^4^ (Ω·m)^−1^. Nitrogen annealing alone substantially reduces the carrier concentration due to vacancy rearrangement, thereby increasing the Seebeck coefficient (*S*), but it limits the σ. In contrast, Se-rich annealing restores near-stoichiometric composition (Se/Bi of 1.57), maintaining adequate carrier density while improving structural coherence of the films. As a result, the 70 nm film annealed in N_2_ + Se exhibits an electrical conductivity (σ) of 1.5 × 10^4^ (Ω·m)^−1^ and *S* = −117 μV K^–1^ at 300 K, yielding a maximum *PF* of 206 μW m^–1^ K^–2^. This value represents nearly 2 orders of magnitude improvement over the as-deposited films, and it compares well with the values recently reported for textured or postdeposition selenized Bi_2_Se_3_ thin films as described in the following examples. Ao et al.^11^ reported a Seebeck coefficient greater than 110 μV K^–1^ with a *PF* of 241 μW m^–1^ K^–2^ from 300 K to 349 μW m^–1^ K^–2^ at 573 K for films prepared by radiofrequency magnetron sputtering. Peng et al.^20^ reported a Seebeck coefficient above 94 μV K^–1^ with a power factor of 127 μW m^–1^ K^–2^ at 350 K in thin films obtained by radio frequency (RF) Magnetron sputtering. More recently, Gao et al.^21^ reported Bi_2_Se_3_ thin films subjected to a 120 min selenization treatment using pulsed laser deposition (PLD), achieving a maximum power factor of 950 μW m^–1^ K^–2^ at 475 K with a *S* coefficient of 115.3 μV K^–1^.

The thinner films (45–57 nm) exhibit higher *S*-coefficient values (−138 to −156 μV K^–1^), consistent with reduced carrier density and confinement effects, but their lower conductivity limits the power factor to 30–83 μW m^–1^ K^–2^. These results indicate that the 70 nm VTE film heated in N_2_–Se ambient provides an optimal balance between carrier concentration, mobility, and texture-enhanced transport.

Overall, the selenium-assisted annealing establishes a direct structure–transport correlation: increasing *TC*(006) from 5.5 to 8.2 and doubling crystallite size result in a 6× enhancement in mobility and an 80× increase in the power factor. This scalable VTE + postselenization strategy provides an effective pathway toward high-performance room-temperature Bi_2_Se_3_ thin-film thermoelectrics.

Figure 9 shows a proof-of-concept thermoelectric Bi_2_Se_3_ thin film thermoelectric generator device prepared by interconnecting five thin film strips of 0.5 cm in width and 2 cm in length, as represented schematically in Figure 9a. Thin films of Bi_2_Se_3_, 45 and 70 nm in thickness, and heated in an N_2_ + Se ambient at 300 °C, were used to make these devices. These devices produced a voltage of 0.7 mV K^–1^, without showing a clear distinction between those made with the 45 and 70 nm films. The measured internal resistance of the device with a 45 nm film was 630 kΩ, and that of the device with a 70 nm film was 540 kΩ. Figure 9b illustrates that the device made with the 70 nm thin film had a response of 0.07 mV s^–1^ or 0.1 K s^–1^ when exposed to solar radiation of approximately 1000 W m^–2^ in intensity.

**Figure 9:**
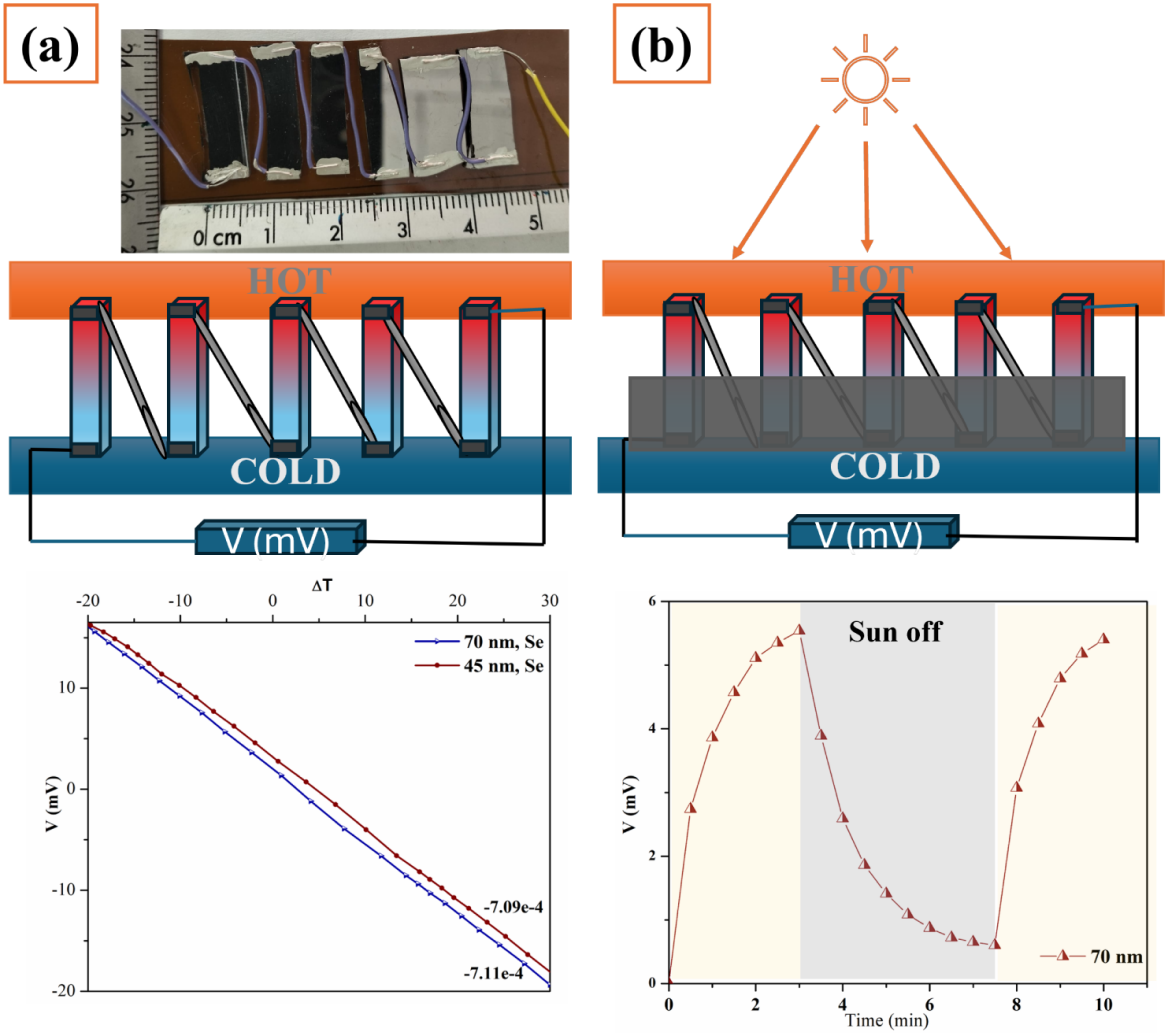
(a) Schematic of five series-connected thermoelectric elements of 0.5 cm in width and 2 cm in length of 45 or 70 nm Bi_2_Se_3_ thin films heated in selenium ambient and the thermo-emf plotted against differential temperature applied across the elements; (b) the thermoelectric generator placed under the sun with one side of the elements blocked from the sun at time *t* = 0 and the sun blocked-off at *t* = 3 min and opened again at 7.5 min (*created by the authors*).

## 4 Conclusions

Thin films of Bi_2_Se_3_ were successfully deposited by vacuum thermal evaporation and further optimized through selenium-assisted postdeposition annealing, leading to significant improvements in their structural, optical, and thermoelectric properties. This annealing effectively compensated for selenium vacancies and improved morphology, thereby enhancing carrier mobility by reducing grain-boundary barriers. Structural characterization confirmed a strong *c*-axis preferential orientation and an increase in crystalline grain diameter from 7 to 17 nm, with an optical band gap ranging from 0.94 to 1.69 eV depending on film thickness. Thermoelectric measurements demonstrated a substantial performance enhancement, with a maximum power factor of 206 μW m^–1^·K^–2^ achieved for a 70 nm film annealed in a selenium ambient. The effectiveness of combining VTE deposition with selenium-assisted thermal processing to obtain high-performance Bi_2_Se_3_ thin films for thermoelectric applications was established. A proof-of-concept thin-film thermoelectric generator using a five-element series-connected 45- or 70 nm Bi_2_Se_3_ thin film was demonstrated. It produced a voltage of 0.7 mV K^–1^ and showed a response of 0.07 mV s^–1^ or 0.1 K s^–1^ when exposed to solar radiation of intensity 1000 W m^–2^.

## Supplementary Material



## Notes

Characterization data of the nanomaterial precursor; experimental setup of the ICP-OES analysis; EDS results; XPS survey spectra of the Bi_2_Se_3_ thin films as-deposited; nitrogen-annealed; selenium-rich atmosphere-annealed by vacuum thermal evaporation; depth profile analysis for Bi and Se; XRD spectra and tables; optical reflectance (R); optical transmittance (T); R + T for Bi_2_Se_3_; optical absorption coefficient (α); (αhν) versus photon energy (hν) plots for thin films deposited by vacuum thermal evaporation (PDF)
