# Supplementary material for: Thermoelectric Characteristics of Bismuth Selenide Thin Films Prepared by Vacuum Thermal Evaporation of Bismuth Selenide Nanoparticles Synthesized by Hot Injection
Source: ACS Omega. 2026 Jun 26;11(27):40079–91. doi: 10.1021/acsomega.6c01937 (PMC13382660; doi:10.1021/acsomega.6c01937)
Supplement: Supplementary file 1 [file ao6c01937_si_001.pdf]

Supporting Information

Thermoelectric characteristics of bismuth selenide thin films prepared by vacuum thermal evaporation of bismuth selenide nanoparticles synthesized by hot injection

Claudia Patricia Villamizar Caballero, Angélica Lizbeth Espinosa Santana., Santhamma Maileppallil Thankamma Nair*, Padmanabhan Karunakaran Nair*

Instituto de Energías Renovables, Universidad Nacional Autónoma de México

Temixco, Morelos – 62580, México. *mtsn@ier.unam.mx

1. Precursor

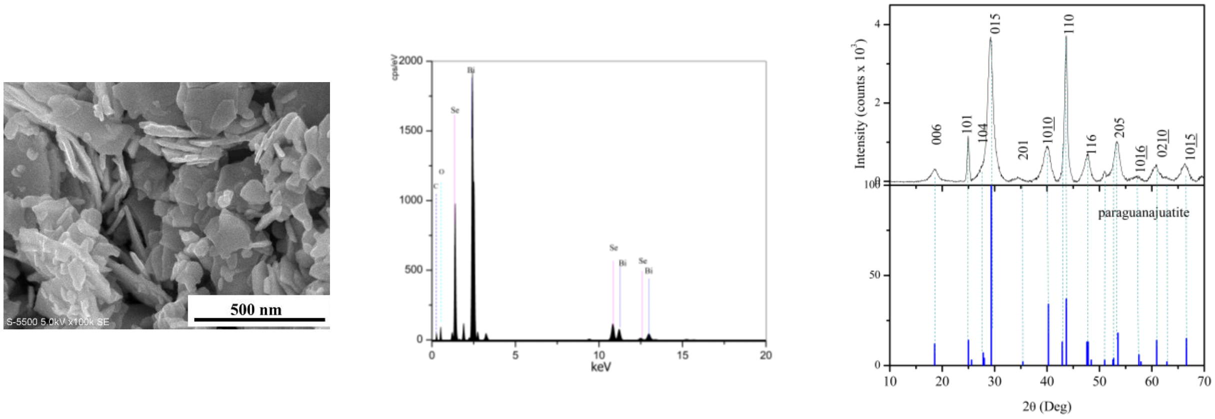

(a) (b) Bi:Se 1:1.53 (c)

Figure S1. (a) SEM images of Bismuth selenide nanosheet used as a precursor for the deposition of thin films by vacuum thermal evaporation (b) EDS spectra and (c) XRD pattern of bismuth selenide NPs. The number showed over the peaks correspond to the miller indices (*hkl*) of the paraguanajuatite mineral structure (trigonal, PDF#00-033-0214).

2. EDS data

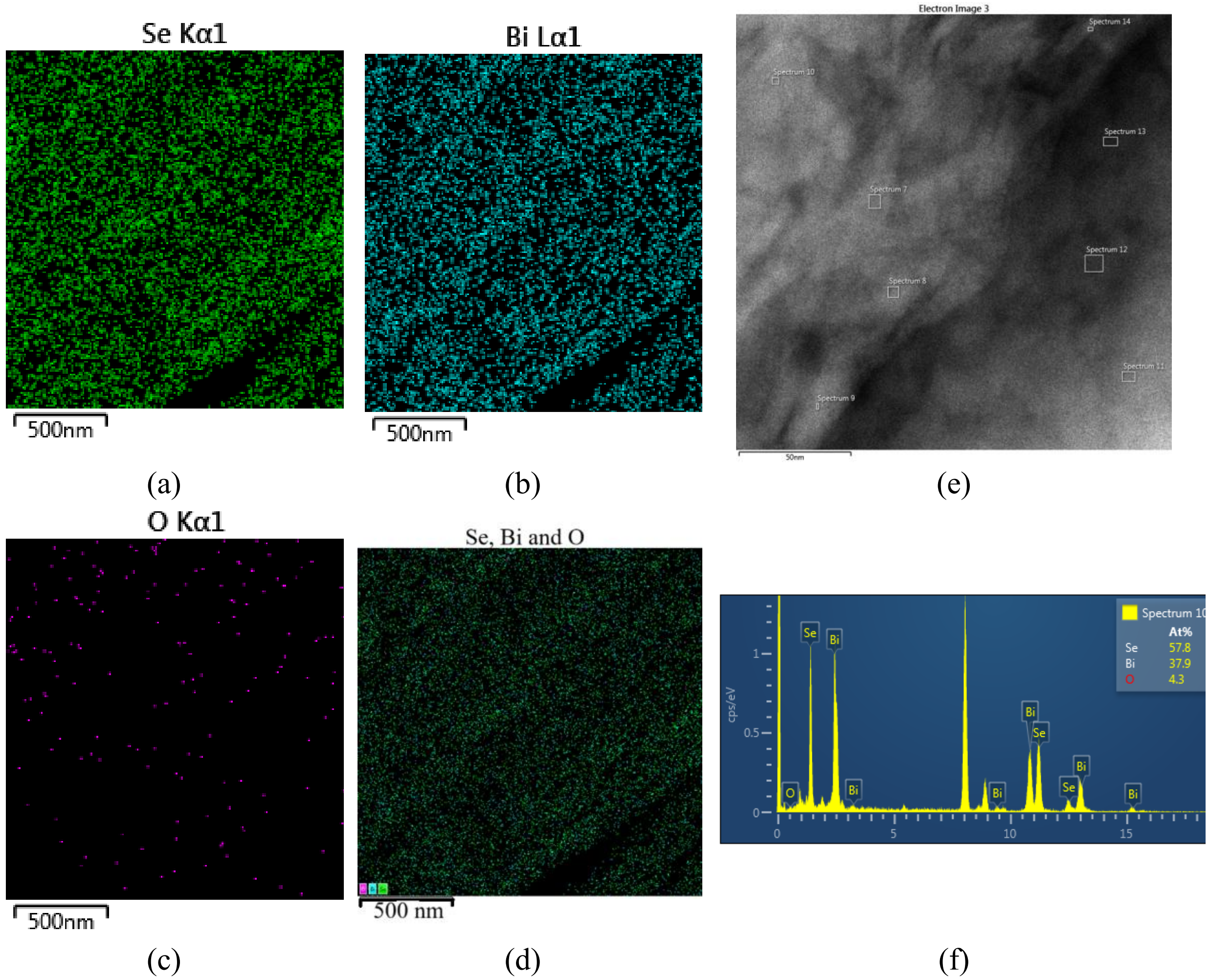

Figure S2. Elemental distribution maps of (**a**) Se $K\alpha_1$, (**b**) Bi $L\alpha_1$, and (**c**) O $K\alpha_1$, (**d**) combined Se, Bi, and O elemental mapping, and (**e**) the sampling micrography for the corresponding EDS analysis and (f) EDS spectrum of a Bi_2Se_3 scrapped-off thin film (70 nm) deposited by vacuum thermal evaporation (VTE) and subsequently annealed in a selenium-rich atmosphere (5 mg Se, 10 torr N_2) for 30 minutes. The emission peaks at 0.930 and 8.047 keV belong to the copper mesh on which the scrapped-off 70 nm VTE thin film was supported.

3. ICP-OES analysis

3.1 Methodology

a) Calibration solution preparation

The solutions used for the calibration curve were prepared from commercially available standard solutions of elements with 1000 mg/liter in 4 % HNO_3, supplied by Crescent Chemical Company. In the present work a stock solution was prepared by mixing 5 cm^3 of Bi solution and 7.5 cm^3 of Se solution in a 50 cm^3 volumetric flask and making the volume up to the mark by the addition of 4 % HNO_3 solution. From this, different volumes taken to have 0. 1, 2, 5, 7, or 10 mg/dm^3 of Bi and 0, 1.5, 3, 7.5, 10.5 or 15 mg/dm^3 of Se in the solution for analysis in order to make the calibration curve.

b) Sample preparation and analysis

Solutions for ICP-OES analysis were prepared from 57 nm Bi_2Se_3 thin films according to the following procedure. A thin film sample of ~3 cm^2 piece of the Bi_2Se_3 on glass substrate was was placed face up in a 50 mL beaker, and 1.52 mL of concentrated nitric acid solution was added dropwise. The thin film was dissolved completely in the acid. Drops of DI water was added to this, and the entire solution was transferred carefully to a 50 cm^3 volumetric flask. The content was transferred completely by rinsing the beaker several times with small quantities of DI water and transferring the solution every time to the flask. Subsequently, the solution was diluted to a total volume of 50 mL by the addition of deionized water to the flas. A 4% HNO_3 solution in DI water was used as a blank. Each element was measured three times per sample. The emission lines of Bi at 223.06 nm and of Se at 196.026 nm were used for the analysis. The methodology selected considered some references that had been analyzed in thin films.[1–3]

Table S1. Working conditions for the ICP-OES spectrometer.

Spectrometer	Horiba-Scientific Ultima 2
Wavelength	Bi 223.06 nm
	Se 196.026 nm
Entrance slit width	0.020 mm
Exit slit width	0.015 mm
Sample uptake rate	2.5 mL/minute
Nebulization pressure	1.0 bar
Auxiliary gas flow rate	Not used
Measurement mode	Gaussian function
Sheath gas stability time	15.0s
Nebulization flowrate	0.02

3.2 Calibration Data

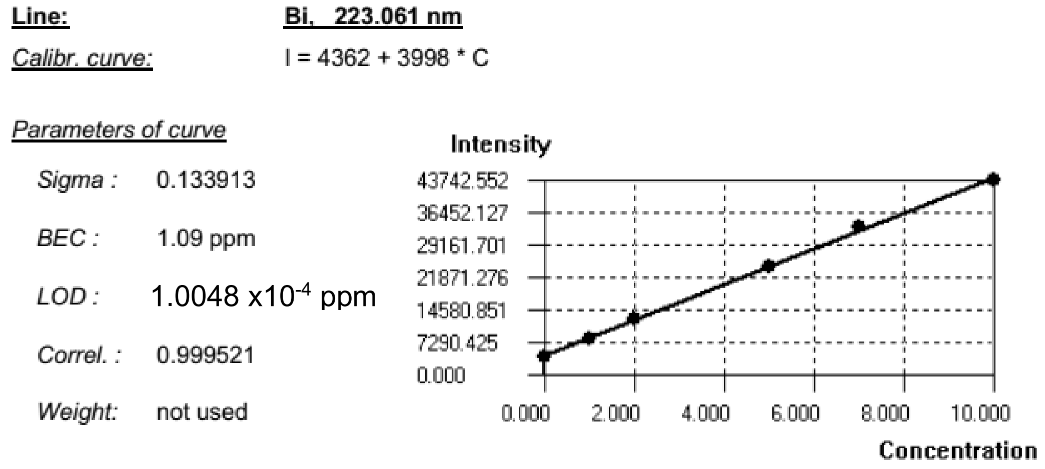

(a)

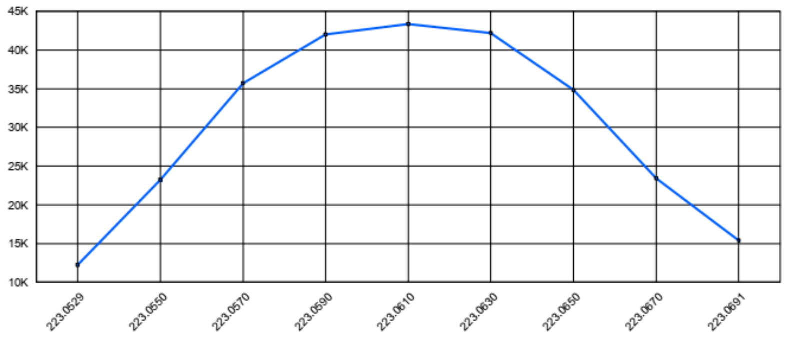

(b)

Line: **Se, 196.026 nm**

Calibr. curve: I = 2583 + 3368 * C

Parameters of curve

Sigma :	0.143955
BEC :	767 ppb
LOD :	1.282 $x10^{-4}$ ppm
Correl. :	0.999754
Weight:	not used

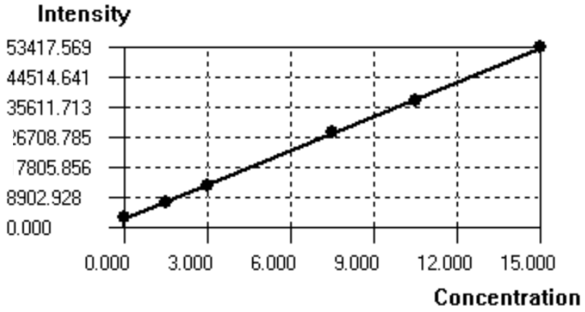

(c)

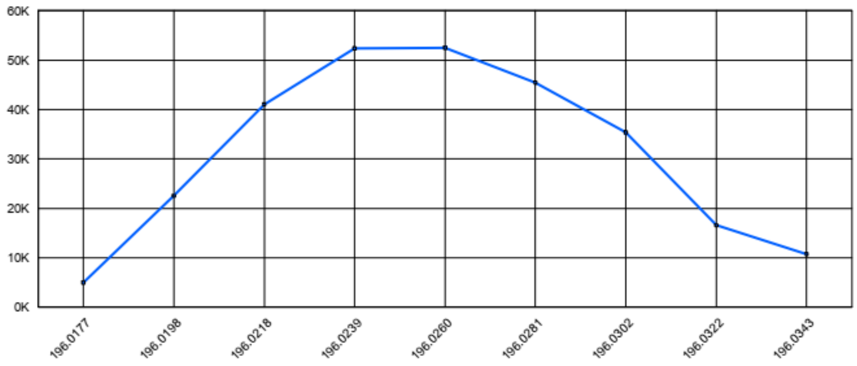

(d)

Figure S3. ICP-OES calibration lines (**a-b**) Calibration lines and experimental data spectrum for Bismuth analyses at the wavelengths 223.061 nm; (**c-d**) calibration lines and experimental data spectrum for Se analyses at the wavelengths 196.026.

3.3 Relative atomic percentage Se: Bi

Table S2. Analytical results reported as Se/Bi ratios obtained from Bi_2Se_3 thin films digested in 4% HNO_3 solution.

Sample	Element	concentration mg L^{-1}	Atomic mass (g mol^{-1})	moles	At, %	Bi: Se ratio	RSD (%)
57 nm	Bi	0.779±0.03	208.98	$3.73x10^{-6}$	39.9	1:1.51	3.4
	Se	0.444±0.02	78.96	$5.62x10^{-6}$	60.1		5.12
57 nm, N_2	Bi	0.900±0.03	208.98	$4.31x10^{-6}$	44.5	1:1.25	2.81
	Se	0.425±0.02	78.96	$5.38x10^{-6}$	55.5		3.96
57 nm, Se	Bi	1.101±0.04	208.98	$5.27x10^{-6}$	35.9	1:1.79	4.53
	Se	0.744±0.05	78.96	$9.42x10^{-6}$	64.1		4.16

4. XPS analysis

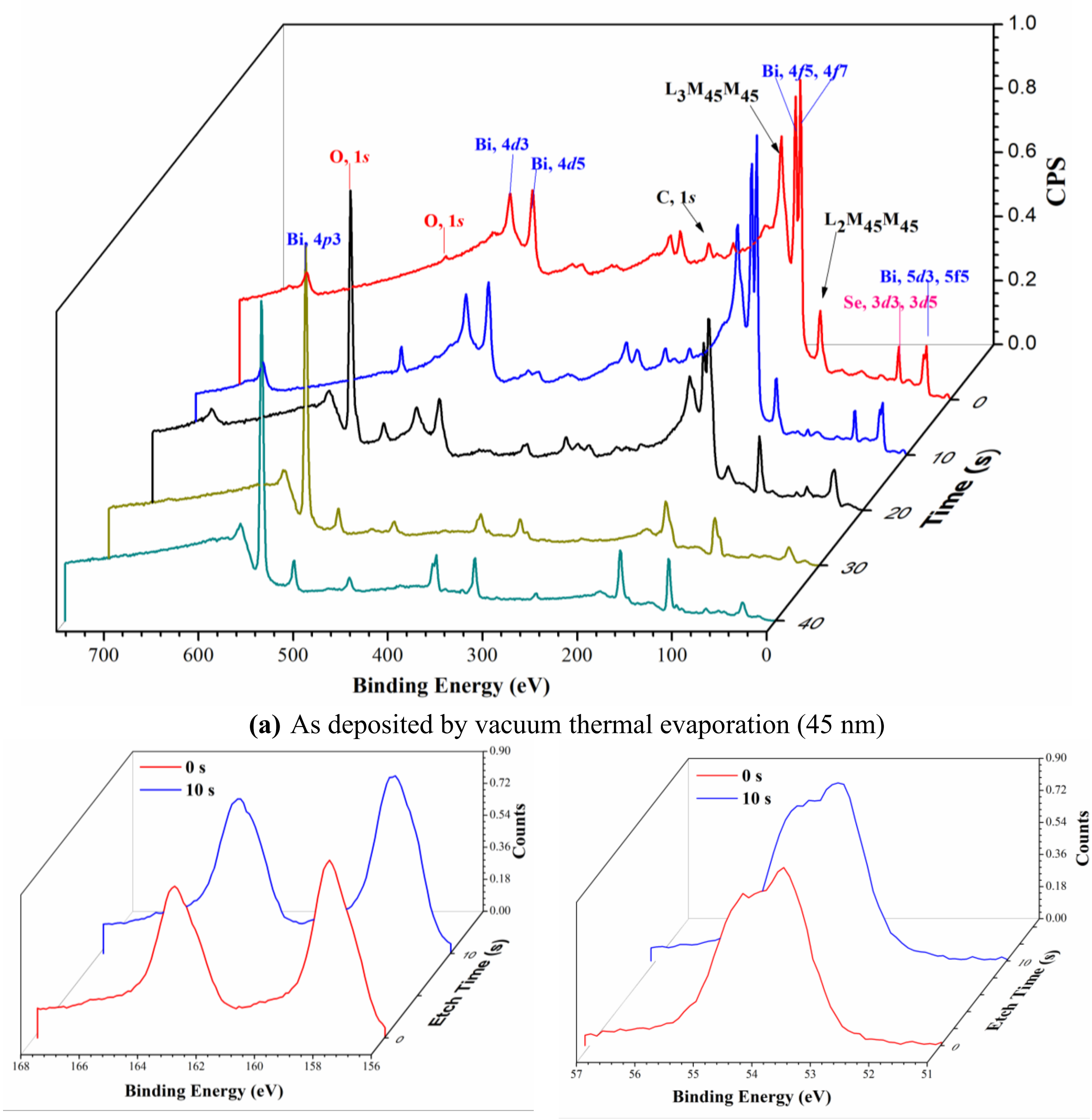

(a) As deposited by vacuum thermal evaporation (45 nm)

(b) (c)

Figure S4. (**a**) XPS survey spectra of the Bi_2Se_3 thin film (45 nm) deposited by vacuum thermal evaporation; (**b–c**) depth profile analysis for Bi and Se, respectively.

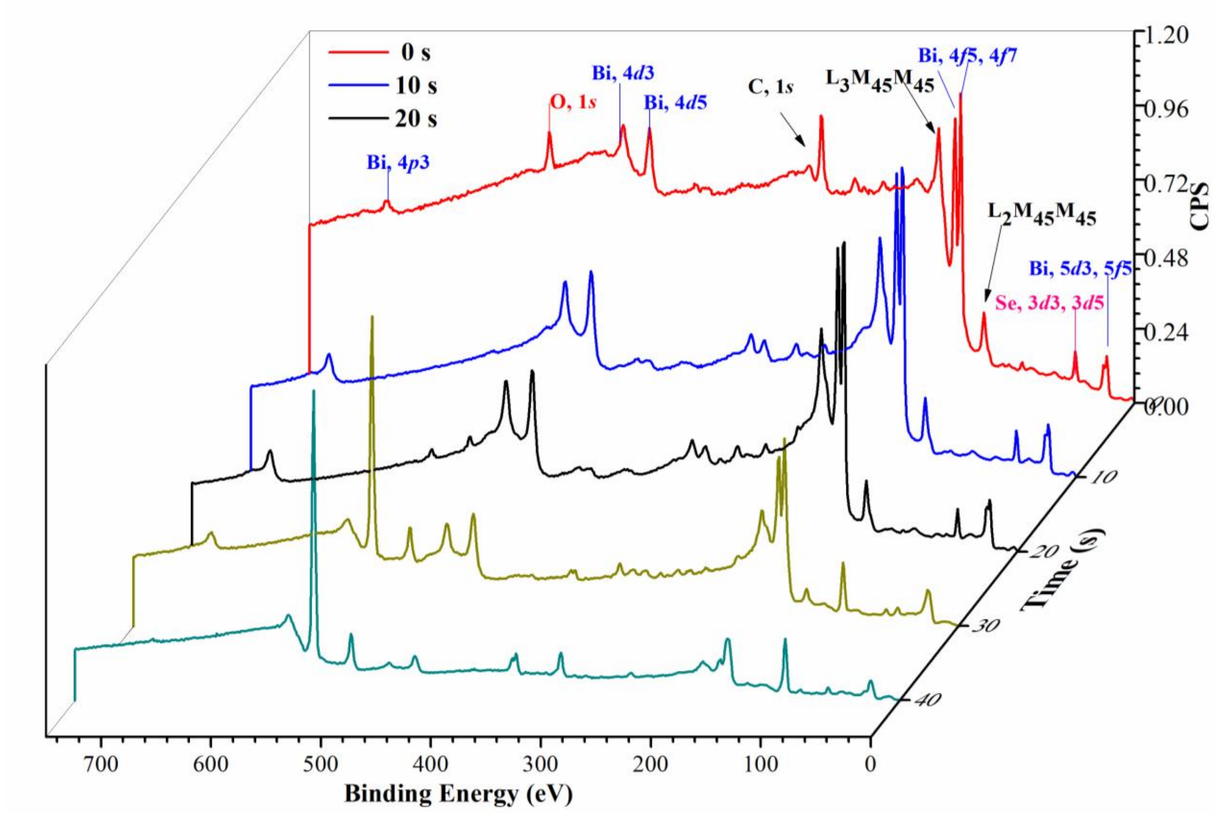

(a) Annealed in N_2 atmosphere (10 torr, 30 min)

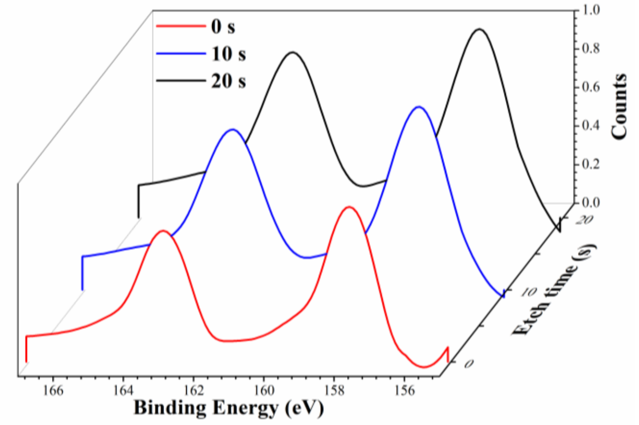

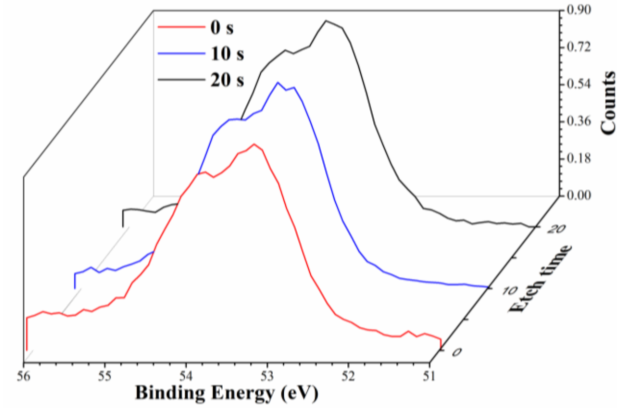

(b) **(c)**

Figure S5. (**a**) XPS survey spectra of the Bi_2Se_3 thin film (45 nm) after the heating in a nitrogen atmosphere (10 torr, 30 minutes); (**b–c**) Depth profile analysis of Bi and Se, respectively.

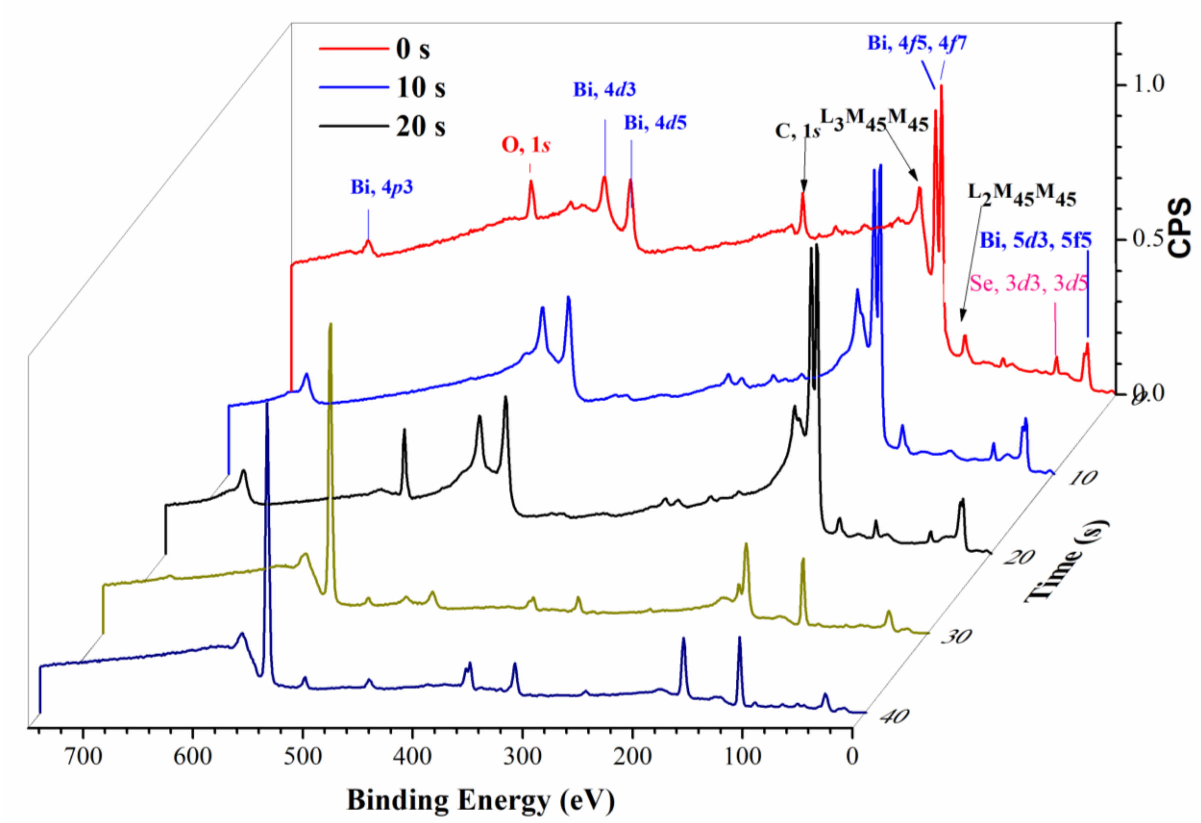

(a) Annealed in Selenium reach atmosphere and N_2 (5 mg, 10 torr, 30 minutes)

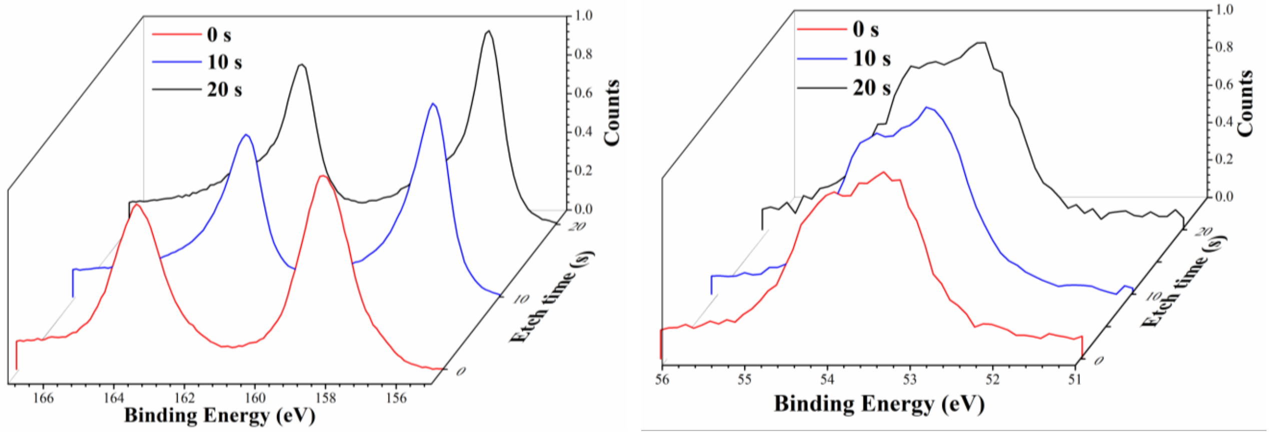

(b) **(c)**

Figure S6. (a) XPS survey spectra of a Bi_2Se_3 thin film (45 nm) prepared by vacuum thermal evaporation and annealed in a selenium-rich nitrogen atmosphere (5 mg Se, 10 torr, 30 minutes); (**b**–**c**) depth profile analysis of Bi and Se, respectively.

5. **Morphology and Crystal structure**

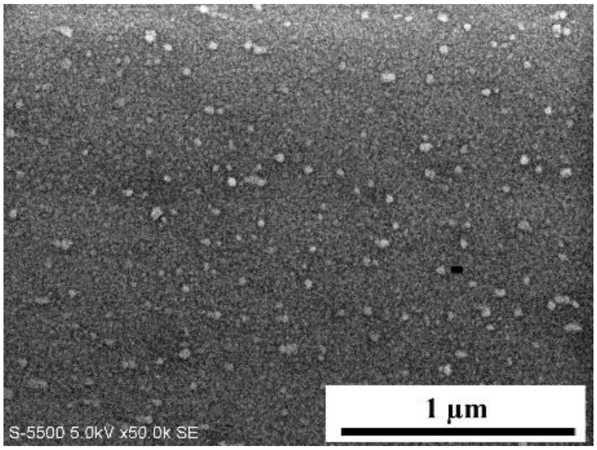

(a) 45 nm film, as deposited by VTE

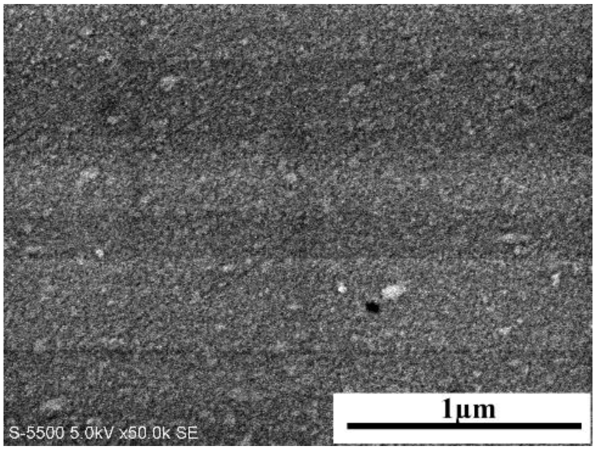

(d) 57 nm, as deposited by VTE

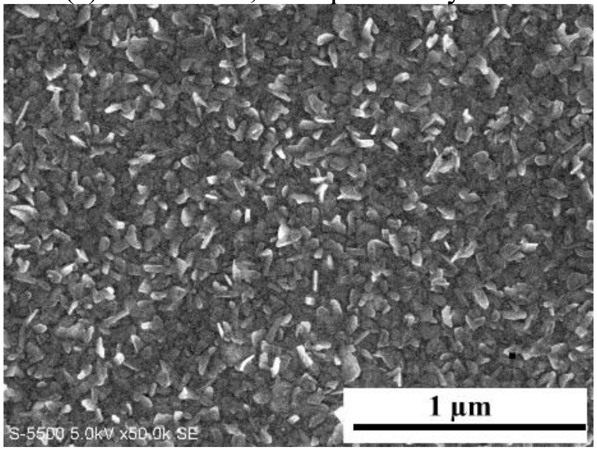

(b) 45 nm, N_2 (10 torr), 300 ºC, 30 minutes

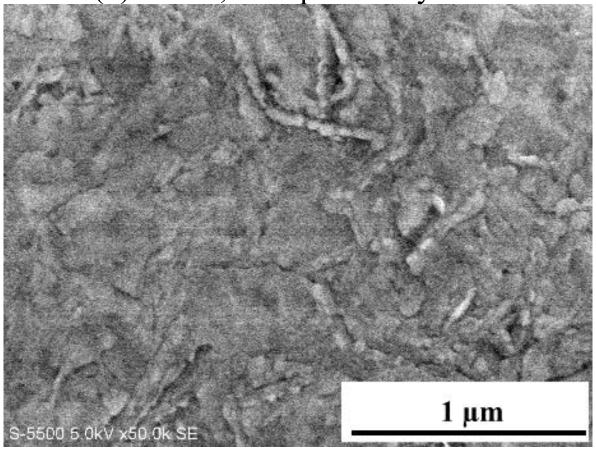

(e) 57 nm, N_2 (10 torr), 300 ºC, 30 minutes

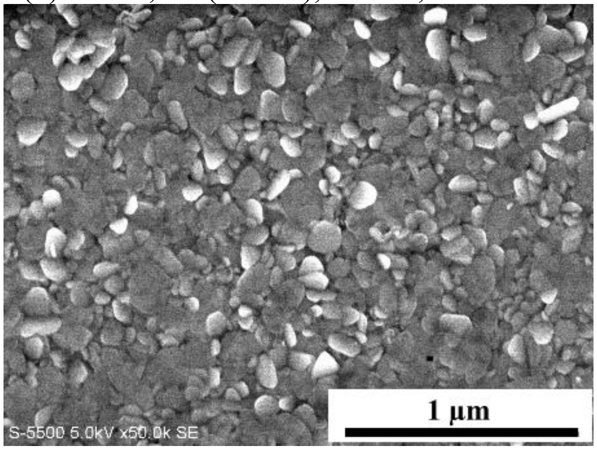

(c) 45 nm, 5 mg Se (powder), N_2 (10 torr), 30 minutes

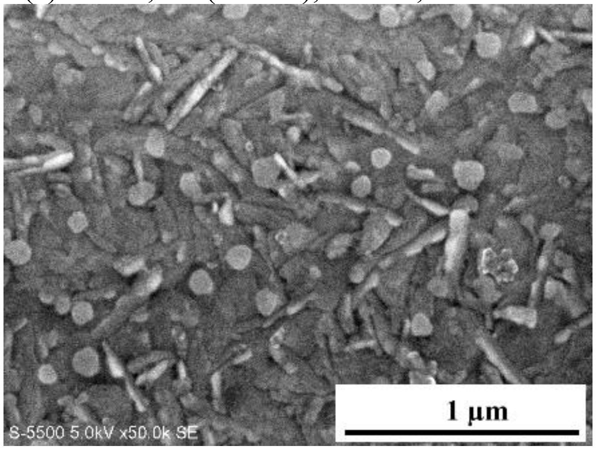

(f) 57 nm, 5 mg Se (powder), N_2 (10 torr), 30 minutes

Figure S7. SEM images of the Bi_2Se_3 thin films by VTE with a thickness 45 nm (a-c) and 70 nm (d-f).

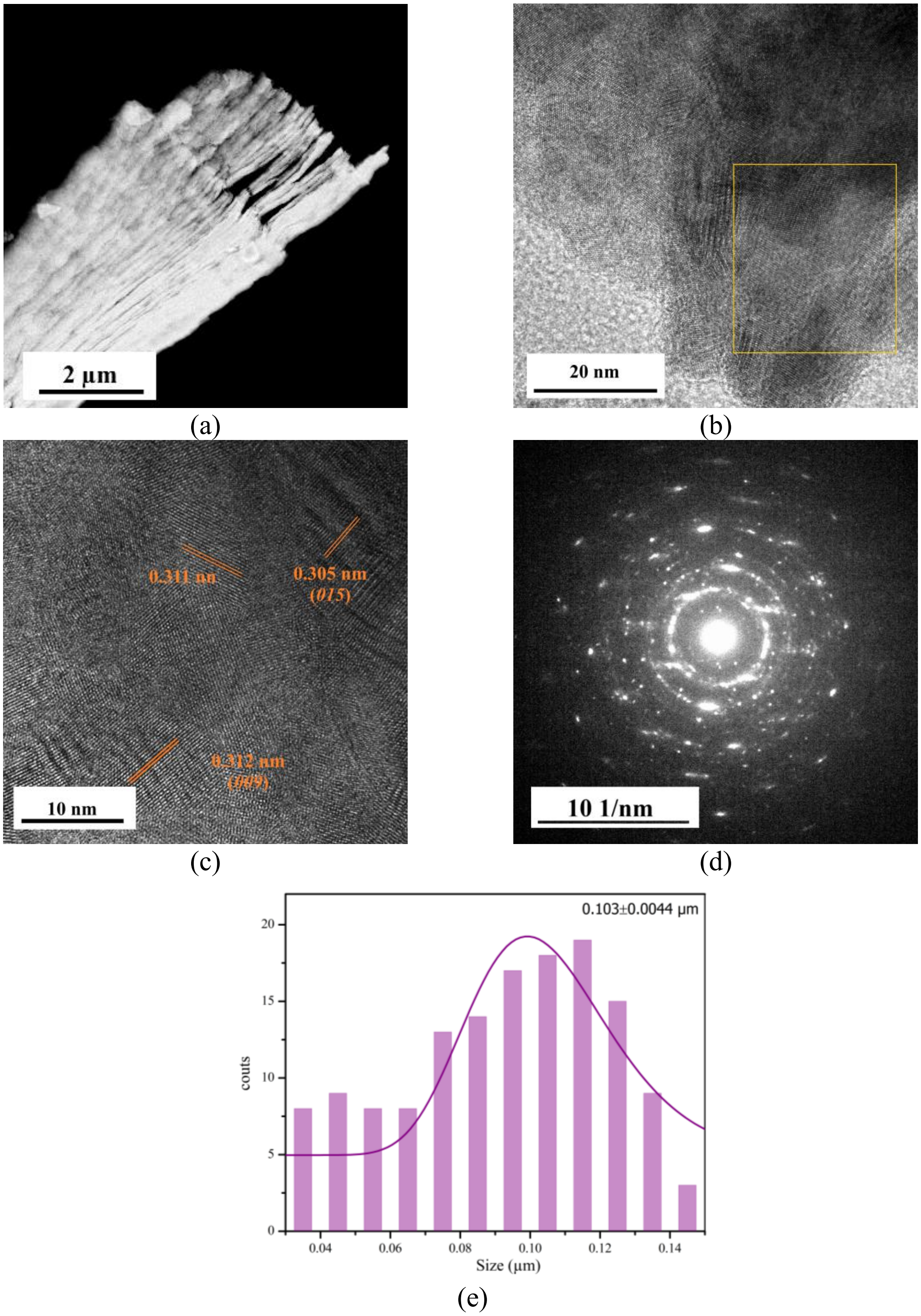

Figure S8. (a) TEM image of Bi_2Se_3 thin film (70 nm, VTE) after thermal treatment in a Se-rich atmosphere, (b-c) HRTEM image of the Bi_2Se_3 thin film (70 nm, VTE), (d) SAED pattern of bismuth selenide thin film, (e) Histogram of particle sizes obtained from SEM images.

6. XRD analysis

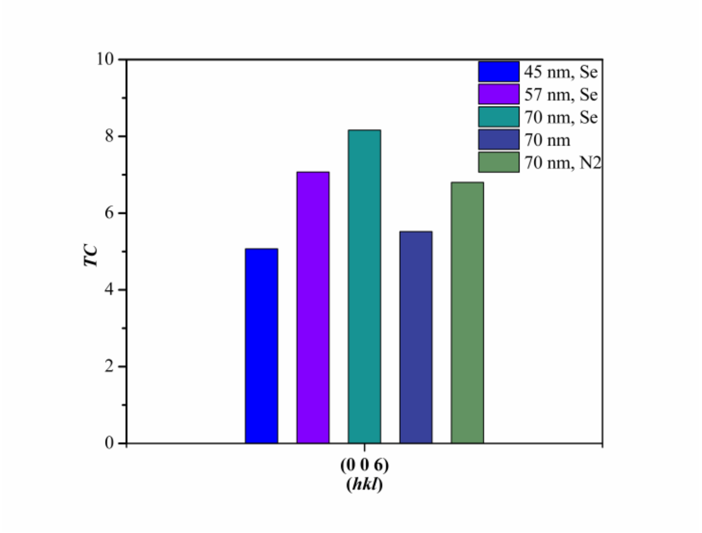

(a)

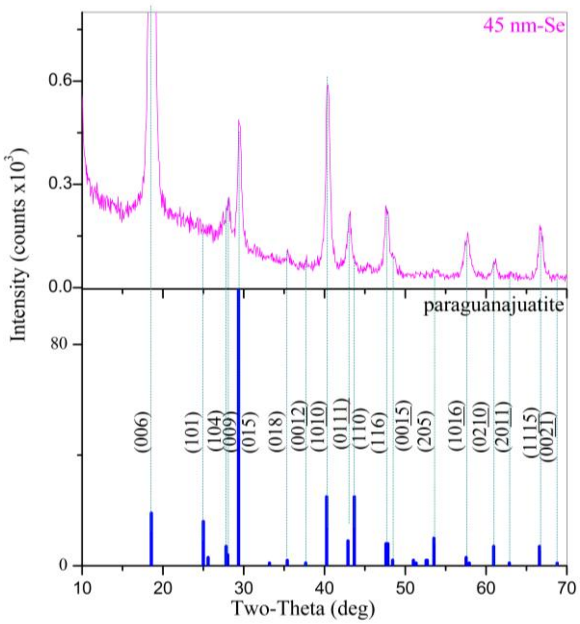

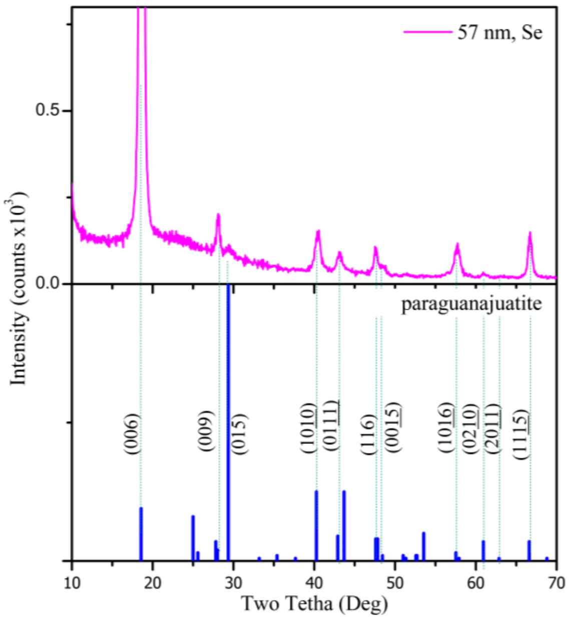

(b) Crystal size (Å): 155

(c) Crystal size (Å): 150

Figure S9. (a) Histogram of texture coefficient of the thin film deposited by vacuum thermal evaporation (45, 57 and 70 nm) for the plane (*006*), (b) XRD pattern for bismuth selenide thin film deposited by VTE (45 nm, Se) and (c) 70 nm, Se.

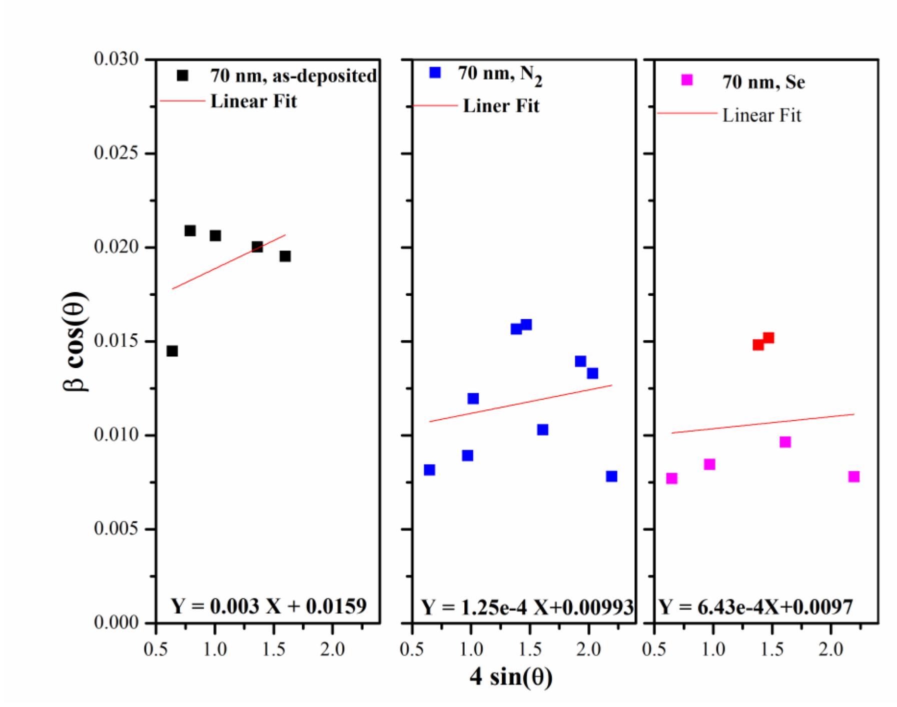

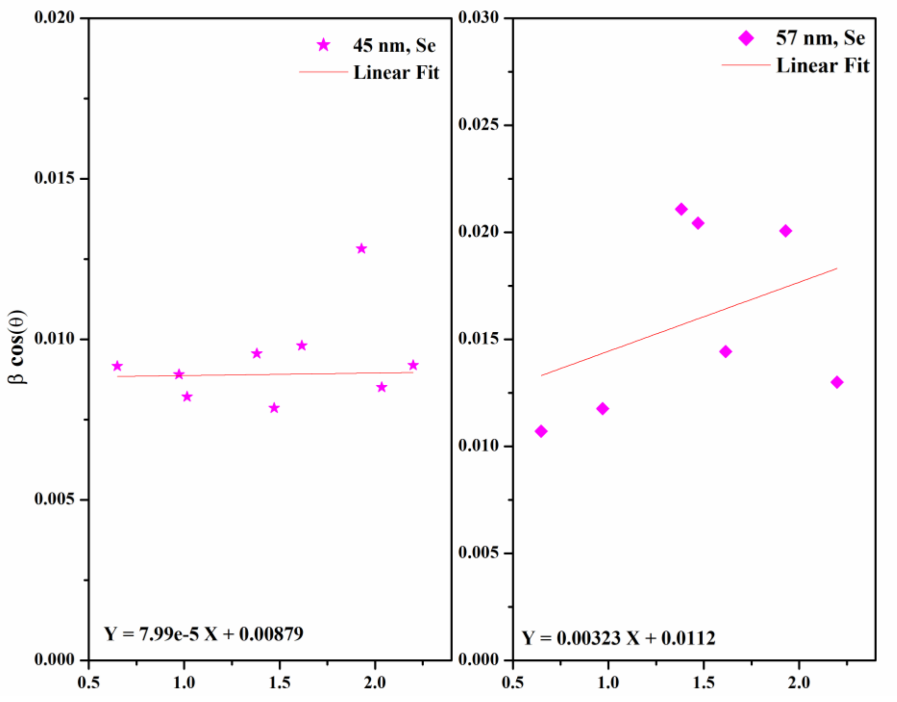

Figure S10. Willimson-Hall plot for Bi_2Se_3 thin films prepared by VTE.

Table S3. Crystallite size (D) calculated by the Scherer equation and Williamson-Hall method, and *d*-spacing for every sample

	2θ(deg)	d(Å)	*hkl*	β(deg)	4*sin(θ)	β*cos(θ)	Ds (nm)	$D_{D\text{-}S}$ (nm)	$D_{W\text{-}H}$ (nm)
**70 nm**	18.4	4.82	0 0 6	0.84	0.64	1.6E-02	99.90	77.16	91.08
	22.9	3.89	0 1 5	1.22	0.79	2.1E-02	69.32		
	29.2	3.06	1 0 10	1.22	1.01	2.1E-02	70.21		
	39.9	2.26	1 1 0	1.22	1.36	2.0E-02	72.27		
	47.1	1.93	0 0 15	1.22	1.60	1.9E-02	74.12		
**70 nm, N_2**	18.6	4.8	0 0 6	0.47	0.65	8.2E-03	177.6	131.5	146.3
	28.1	3.2	0 0 9	0.53	0.97	8.9E-03	162.2		
	29.5	3.0	0 1 5	0.71	1.02	1.2E-02	121.1		
	40.5	2.2	1 0 10	0.96	1.38	1.6E-02	92.5		
	43.1	2.1	1 1 0	0.98	1.47	1.6E-02	91.1		
	47.4	1.9	0 0 15	0.64	1.61	1.0E-02	140.6		
	57.7	1.6	1 0 16	0.91	1.93	1.4E-02	103.8		
	61.1	1.5	0 2 10	0.88	2.03	1.3E-02	108.9		
	66.6	1.4	1 1 15	0.54	2.19	7.8E-03	185.4		
**70 nm, Se**	18.6	4.8	0 0 6	0.45	0.65	7.7E-03	188.0	173.7	178.8
	28.1	3.2	0 0 9	0.50	0.97	8.5E-03	171.2		
	47.5	1.9	1 1 6	0.60	1.61	9.6E-03	150.1		
	66.6	1.4	1 1 15	0.54	2.20	7.8E-03	185.5		
**57 nm, Se**	18.6	4.8	0 0 6	0.38	0.65	6.6E-03	219.5	155.8	129.3
	28.1	3.2	0 0 9	0.43	0.97	7.3E-03	198.4		
	40.4	2.2	1 0 10	0.81	1.38	1.3E-02	108.7		
	43.1	2.1	1 1 0	0.80	1.47	1.3E-02	112.2		

	47.6	1.9	1 1 6	0.57	1.62	9.1E-03	160.0		
	57.7	1.6	1 0 16	0.83	1.93	1.3E-02	114.2		
	66.7	1.4	1 1 15	0.56	2.20	8.2E-03	177.7		
**45 nm, Se**	18.7	4.7	0 0 6	0.53	0.65	9.2E-03	157.9	157.9	181.0
	28.1	3.2	0 0 9	0.53	0.97	8.9E-03	162.6		
	29.4	3.0	0 1 5	0.49	1.01	8.2E-03	176.3		
	40.4	2.2	1 0 10	0.58	1.38	9.6E-03	151.5		
	43.2	2.1	1 1 0	0.48	1.47	7.9E-03	184.2		
	47.7	1.9	0 0 15	0.61	1.62	9.8E-03	147.7		
	57.7	1.6	1 0 16	0.84	1.93	1.3E-02	112.9		
	61.2	1.5	0 2 10	0.57	2.03	8.5E-03	170.1		
	66.7	1.4	1 1 15	0.63	2.20	9.2E-03	157.5		

Table S4. The characteristics of the XRD peaks, strain, dislocations, and *SF*

	**2θ(deg)**	**ε**	**Average (ε) x 10^{-2}**	**δ (nm^{-2})**	**(SF)**	**Average (SF) x 10^3**
**70 nm**	18.4	9.06E-02	7.71 ±2.30	1.00E-04	5.33E-03	5.65 ± 0.87
	22.8	1.05E-01		2.08E-04	6.93E-03	
	29.1	8.20E-02		2.03E-04	6.11E-03	
	39.8	5.88E-02		1.91E-04	5.18E-03	
	47.1	4.89E-02		1.82E-04	4.72E-03	
**70 nm, N_2**	18.6	5.0E-02	3.52 ±1.21	3.2E-05	3.0E-03	3.04 ±0.73
	28.1	3.7E-02		3.8E-05	2.7E-03	
	29.5	4.7E-02		6.8E-05	3.5E-03	
	40.5	4.5E-02		1.2E-04	4.0E-03	
	43.1	4.3E-02		1.2E-04	4.0E-03	
	47.4	2.6E-02		5.1E-05	2.5E-03	
	57.7	2.9E-02		9.3E-05	3.1E-03	
	61.1	2.6E-02		8.4E-05	2.9E-03	
	66.6	1.4E-02		2.9E-05	1.7E-03	
**70 nm, Se**	18.6	4.8E-02	3.03 ±1.46	2.8E-05	2.8E-03	2.33 ±0.47
	28.1	3.5E-02		3.4E-05	2.5E-03	
	47.5	2.4E-02		4.4E-05	2.3E-03	
	66.6	1.4E-02		2.9E-05	1.7E-03	
**57 nm, Se**	18.6	4.2E-02	3.01 ± 0.95	2.1E-05	2.5E-03	2.63 ±0.59
	28.1	3.1E-02		2.5E-05	2.3E-03	
	40.4	3.9E-02		8.5E-05	3.4E-03	
	43.1	3.5E-02		7.9E-05	3.3E-03	
	47.6	2.3E-02		3.9E-05	2.2E-03	

	57.7	2.6E-02		7.7E-05	2.9E-03	
	66.7	1.5E-02		3.2E-05	1.8E-03	
**45 nm, Se**	18.7	5.8E-02	2.93±1.26	4.0E-05	3.4E-03	2.48± 0.46
	28.1	3.7E-02		3.8E-05	2.7E-03	
	29.4	3.3E-02		3.2E-05	2.5E-03	
	40.4	2.8E-02		4.4E-05	2.5E-03	
	43.2	2.2E-02		2.9E-05	2.0E-03	
	47.7	2.5E-02		4.6E-05	2.4E-03	
	57.7	2.7E-02		7.8E-05	2.9E-03	
	61.2	1.7E-02		3.5E-05	1.9E-03	
	66.7	1.7E-02		4.0E-05	2.0E-03	

Table S5. SAED, XRD, and HRTEM data for the Bi_2Se_3 thin film (57 nm) deposited by vacuum thermal evaporation (VTE) and annealed at 300 °C for 30 min in a selenium-rich atmosphere (5 mg Se, 10 torr of nitrogen); r is the radius of interference fringe.

**SAED**					**PDF#00-33-0214**		**XRD**	**HRTEM**
No.	1/2r (nm^{-1})	1/r (nm^{-1})	r (nm)	d-spacing (Å)	(*hkl*)	d-(Lit) (Å)	d-(Exp) (Å)	d-(Exp) (Å)
1	5.5	2.6	0.36	3.64	*(1 0 1)*	3.56	3.58	
2	6.4	3.2	0.31	3.12	*(0 0 9)*	3.18	3.16	0.313
3	8.6	4.3	0.23	2.33	*(0 0 12)*	2.39	2.37	
4	9.3	4.7	0.21	2.14	*(0 1 11)*	2.11	2.1	
5	10.1	5.1	0.19	1.98	*(1 1 0)*	2.1	2.08	0.190
6	11.4	5.7	0.16	1.75	*(2 0 5)*	1.71	1.72	
7	12.8	6.4	0.16	1.56	*(0 2 10)*	1.52	1.52	

7. Optical

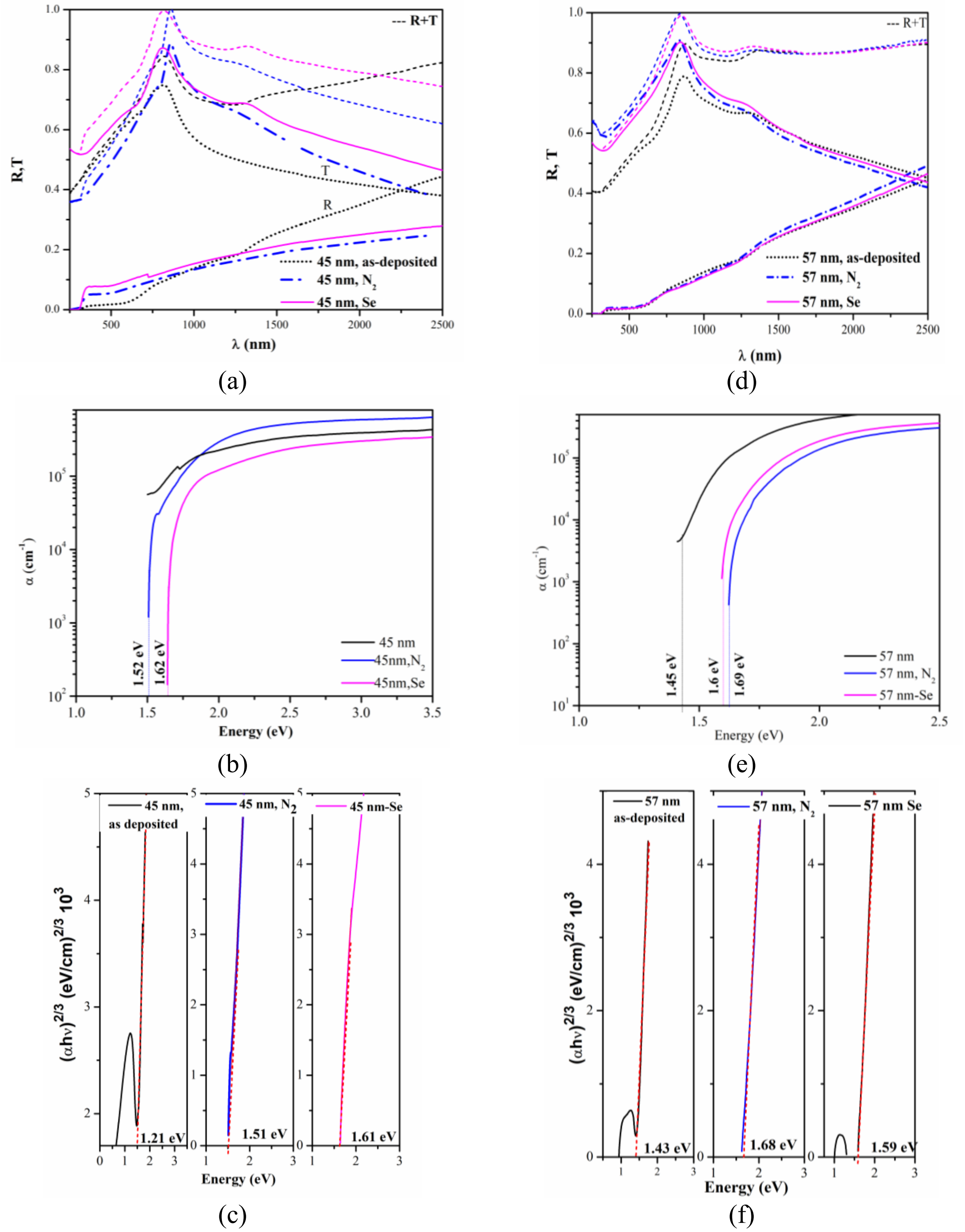

Figure S11. (a-d) Optical reflectance (R) and Optical transmittance (T) and R+T for Bi_2Se_3 thin films deposited by vacuum thermal evaporation with a thickness of 45 nm and 57 nm; (**b, e**) Optical

absorption coefficient (α); and (**c**, **f**) $(\alpha h\nu)^2$ versus photonenergy (hν) plots: black, as prepared thin films; blue, thin films heated at 300 °C under 10 torr of nitrogen for 30 minutes; magenta, for the film heated in Se-vapor at 300 °C.

References

[1] Morrison C, Sun H, Yao Y, Loomis RA, Buhro WE. Methods for the ICP-OES Analysis of Semiconductor Materials. Chem Mater 2020;32:1760–8. https://doi.org/10.1021/acs.chemmater.0c00255.

[2] Fernández-Martínez R, Caballero R, Guillén C, Gutiérrez MT, Rucandio MI. Application of ICP-OES to the determination of $CuIn_{1-x}Ga_xSe_2$ thin films used as absorber materials in solar cell devices. Anal. Bioanal. Chem., vol. 382, 2005, p. 466–70. https://doi.org/10.1007/s00216-004-2997-z.

[3] Perkins CL, Egaas B, Repins I, To B. Quantitative analysis of graded $Cu(Ix_{1-x},Ga_x)Se_2$ thin films by AES, ICP-OES, and EPMA. Appl Surf Sci 2010;257:878–86. https://doi.org/10.1016/j.apsusc.2010.07.085.
